# Quaternary Alloy Quantum Dots as Fluorescence Probes for Total Acidity Detection of Paper-Based Relics

**DOI:** 10.3390/nano11071726

**Published:** 2021-06-30

**Authors:** Zhuorui Wang, Cong Cheng, Yongjuan Cheng, Lizhen Zheng, Daodao Hu

**Affiliations:** 1 Engineering Research Center of Historical Cultural Heritage Conservation, Ministry of Education, School of Materials Science and Engineering, Shaanxi Normal University, Xi’an 710119, China; wangzhuorui@snnu.edu.cn (Z.W.); congcheng2017@snnu.edu.cn (C.C.); chengyongjuan@snnu.edu.cn (Y.C.); 2 School of Historical Culture and Tourism, Xi’an University, Xi’an 710065, China

**Keywords:** total acidity, bound protons, quaternary alloy QDs, fluorescence probe, paper-based relic

## Abstract

Traditionally, the acidity of paper-based relics was determined by an extraction method and using a pH meter. This method could not obtain the total acidity of the paper-based relics because it only detected the concentration of free protons in the aqueous soaking solution. To overcome this defect, a new method for determining the total acidity of paper-based relics has been established by using quaternary alloy quantum dots. The quantum dots, CdZnSeS, modified by p-Aminothiophenol (pATP) were prepared, and their composition and structure were characterized. The fluorescence behavior of prepared quantum dots with acidity was investigated. The following results were obtained. The fluorescence of CdZnSeS-pATP quantum dots could decrease with increases in acidity because pATP dissociated from the surfaces of the quantum dots due to protons or undissociated weak acids. Based on this feature, a method for determining the acidity of paper-based relics was constructed, and this method was used to evaluate the acidity of actual paper-based relics. Obviously, for a given paper sample, since both free protons and bound protons can be determined by this method, the acidity measured by this method is more reasonable than that by pH meter.

## 1. Introduction

Paper-based artifacts are one kind of popular and important historical cultural relics. As one of the primary carriers for culture and art, the paper plays a vital role in the dissemination and inheritance of information. However, acid groups formed by the oxidation of papermaking raw materials, residues from pulping and sizing, acid gases in the atmosphere, and organic acid secreted by mildew can all bring acidity to paper [1]. The β-acetal oxygen bridge joining the glucose molecules of cellulose together, is susceptible to acid hydrolysis which breaks the chains and weakens the fibers. Paper which decomposes this way becomes hard and brittle, and disintegrates easily [2]. For instance, when a paper sample was very acidic (pH = 3.20), its DP (degree of polymerization) dropped from 760 to 250 in only 22 years. On the other hand, when a paper sample had a higher pH value (pH = 5.72), its DP was nearly unchanged after the same period of time [3]. According to the life expectancy equation predicted by kinetic models, when the pH of paper decreases by one unit, the lifetime of paper will reduce by around 10 times [4]. Plenty of paper-based cultural relics should accept deacidification to extend their lifetimes based on this situation. Accordingly, the determination of acidity is of great significance to the protection of paper-based cultural relics. 

The cold extraction method to determine the pH of aqueous extracts of shredded paper by pH meter is based on the definition of paper acidity, which is that surplus [H^+^] are produced by soluble substances in paper [5]. A method in which the flat electrode of a pH meter contacts the wet part of paper is also used to measure the surface pH of paper [6]. The two methods, however, can only respond to free protons in aqueous solutions or in paper, but they cannot detect the protons bound to acidic groups, such as carboxyl or phenolic hydroxyl of hemicellulose and lignin, or the adsorbed protons on cellulose, pectin and the protein in paper. These bound protons have a greater impact on paper degradation than free protons [7]. Therefore, it is necessary to improve the method for evaluating acidity of paper-based relics. If free protons and undissociated acid in paper could react with probe molecules, the sensing of pH values could potentially be more reliable than using a pH meter. In this case, the sum of free and bound proton content reflects the real degree of acidification of paper. For determining the pH of paper-based relics, it is very important to use a non-destructive analysis technology. From this point of view, one advisable strategy is that the paper acidity is determined by fluorescence (FL) probes. Unfortunately, using fluorescence probes to determine paper acidity is scarcely reported in the literature. To our best knowledge, Moorthy et al. [8] used fluorescence probes to estimate paper acidity for the first time. They attempted to prepare paper samples of varying acidity by soaking near-neutral filter paper in buffer solutions with varying pH for almost one day so that an acidity equilibrium might be achieved between the paper and buffer solution. The changes in fluorescence properties of the four probes (quinine bisulphate, fluorescein, pyranine and a seminaphthofluorescein dye) in different pH buffer solutions were found to correlate with the changes in fluorescence properties of the probes applied on paper samples of varying acidity [8]. Although the authors have completed groundbreaking work, the four probes are not practical enough since a narrow pH sensing range of any probe in the assay does not meet the actual range of the pH requirement. In addition, Qu et al. [9] developed a new fluorescence nanosensor to measure the surface pH of paper. This nanosensor is based on chitosan-modified silica nanoparticles. Ru(bpy)_3_^2+^ was doped in the nanoparticles as a reference dye and pH sensitive fluorescein isothiocyanate was used as the pH indicator. The pH nanosensor showed the linear dynamic range from 5.5 to 8.0 [9]. Distinctly, these methods are not suitable to determine the pH of acidic paper-based relics. 

Semiconductor quantum dots (QDs) are inorganic fluorescence nanocrystals with a wide absorption range, narrow emission linewidth, high fluorescence quantum efficiency and excellent chemical stability and photobleaching resistance compared with organic molecules [10,11,12,13]. The quaternary alloy CdZnSeS QDs have a core–shell structure with good photostability [14], and the alloy structure can eliminate defects on interfaces between core and shell caused by lattice mismatch to acquire high fluorescence quantum efficiency [15]. Additionally, CdSe/ZnS core/shell QDs with ligands of p-Aminothiophenol (pATP) could respond to a pH range of 3.2–6.0 in aqueous solution [16]. 

According to the properties of CdSe/ZnS modified by pATP, a nondestructive method with a wide pH sensing range to detect the acidity of paper was established in this paper. The results indicated that this method could be used to determine the total acidity (including free protons, adsorbed protons and undissociated protons) of paper. Unlike determining acidity by a pH meter, adsorbed protons and undissociated protons could be measured by the new method, besides free protons. In fact, the adsorbed protons and undissociated protons have more damaging effects on paper than free protons. Therefore, the new method used to evaluate the acidity of paper-based relics is more reasonable than extraction methods using a pH meter. To our best knowledge, this is the first time that the total acidity of paper has been monitored. Obviously, this method is very significant for the evaluation of paper-based relics in the field of heritage protection. Of course, this method may also be enlightening for total acidity detection in other materials.

## 2. Materials and Methods

### 2.1. Chemicals

Quaternary alloy QDs (CdZnSeS-OA, 50 mg/mL in n-Hexane) were purchased from Guangdong Poly OptoElectronics (Guangzhou, China) and they were used directly without any further purification. p-Aminothiophenol (C_6_H_7_NS, 97%), deuterium chloride (DCl, 20% in D_2_O (D, 99%)) and tetramethylammonium chloride (C_4_H_12_NCl, AR) were purchased from Shanghai Macklin Biochemical (Shanghai, China). Hydrochloric acid (HCl, AR), glacial acetic acid (CH_3_CO_2_H, AR), aluminium potassium sulfate dodecahydrate (KAl(SO_4_)_2_•12H_2_O, AR) and potassium tetraoxalate dihydrate (KH_3_(C_2_O_4_)_2_•2H_2_O, CP) were purchased from Sinopharm Chemical Reagent Co. (Shanghai, China). Sodium hydroxide (NaOH, AR), sodium chloride (NaCl, AR), sodium nitrate (NaNO_3_, AR), sodium sulfate anhydrous (Na_2_SO_4_, AR), gelatin (BC), potassium hydrogen phthalate (C_6_H_4_CO_2_HCO_2_K, AR), potassium dihydrogen phosphate (KH_2_PO_4_, AR) and disodium hydrogen phosphate dodecahydrate (Na_2_HPO_4_•12H_2_O, AR) were purchased from Tianjin Tianli Chemical Reagents (Tianjin, China). Lignin alkali (C_30_H_25_ClN_6_, BR) were purchased from Nanjing Duly Biotechnology (Nanjing, China). Deuterium oxide (D_2_O, D > 99.9%) and dimethyl sulfoxide-d6 (DMSO-d6, D > 99.8%) were purchased from Beijing Chongxi High-Tech Incubator (Beijing, China). All organic solvents with AR grade were obtained from Sinopharm Chemical Reagents. All chemicals were used directly without any further purification. Fresh deionized water was used to prevent CO_2_ pollution.

### 2.2. Preparation of CdZnSeS-pATP QDs

CdZnSeS-pATP QDs were prepared by ligand exchange performed as a modified procedure from the literature [17]. A 5 mL volume of pATP solution (2.5 M in ethanol) was injected into 5 mL of CdZnSeS-OA QDs solution (1 mg/mL in n-Hexane), and then the mixture was stirred in a nitrogen-filled glovebox for 24 h. The product was collected by centrifugation and washed with ethanol several times to remove the excess ligand. The purified CdZnSeS-pATP QDs were dispersed in DMSO or chloroform.

### 2.3. Characterization of CdZnSeS-pATP QDs

XRD measurement was carried out on a Rigaku SmartLab X-ray diffractometer (Rigaku, Tokyo, Japan) operated at 45 kV/200 mA with Cu Kα line (λ = 1.5418 Å). The scan step was 0.01⁰ and the speed was 3⁰/min. The powder of CdZnSeS-pATP QDs was measured after vacuum drying. TEM images and SAED patterns were taken on a JEOL JEM-2800 field emission transmission electron microscope (JEOL, Tokyo, Japan) at 200 kV. The samples were prepared by placing one drop of dilute solution of CdZnSeS-pATP QDs in chloroform onto a carbon film on a copper grid and then volatilizing the remaining solvent. Particle size was analyzed manually by modeling each CdZnSeS QD as a sphere, with statistical analysis performed using Nano Measurer software (1.2.0, Fudan University, Shanghai, China) on populations of 100 counts. The energy dispersive spectroscopy (EDS) mappings were carried out by an EDS detector equipped on a JEOL JEM-2800 TEM. HRTEM images were taken on an FEI Tecnai G2 F20 field emission transmission electron microscope (FEI, Hillsboro, OR, USA).

FT-IR spectra were acquired on a PerkinElmer Spectrum Two FT-IR spectrometer (PerkinElmer, Waltham, MA, USA) with KBr pellets of CdZnSeS QDs after vacuum drying. UV-Vis spectra of solutions were taken on a PerkinElmer Lambda 35 UV/VIS spectrometer (PerkinElmer, Waltham, MA, USA). The slit width was 1 nm and scan speed was 480 nm/min. The quantum yield of the samples were determined using a Hamamatsu C9920-02G (Hamamatsu, Iwata, Japan) quantum efficiency measurement system. The fluorescence lifetime measurement was performed using an Edinburgh FLUORESCENCES1000 (Edinburgh, Livingston, UK) transient state fluorescence spectrometer with a 340 nm pulsed diode laser by time correlated single photon counting. All fluorescence decay curves were measured at a maximum emission wavelength of 537 nm. ^1^H NMR spectra were acquired on a JEOL 400 MHz nuclear magnetic resonance spectrometer (JEOL, Tokyo, Japan). The UV-Vis absorbance measurements of paper samples were carried out in the diffuse reflectance mode, using a PerkinElmer Lambda 1050 UV/VIS/NIR spectrometer (PerkinElmer, Waltham, MA, USA) equipped with a 150 mm integrating sphere. The slit width was 2 nm and the scan speed was 266.75 nm/min.

### 2.4. Preparation of Aqueous Solutions with Standardized pH Values

The aqueous solutions with different standardized pH values were prepared as follows. A series of aqueous solutions with different pH values (1–7) were prepared by HCl or NaOH solution, and their pH values were determined by Mettler Toledo SevenCompact pH meter (Mettler Toledo, Columbus, OH, USA) after calibrations using KH_3_(C_2_O_4_)_2_, C_6_H_4_CO_2_HCO_2_K, KH_2_PO_4_ and Na_2_HPO_4_ standard buffer solutions. The pH for each solution was measured twice, and the difference between the two measurements could not exceed 0.02. The final pH is the average of two measurements [18].

### 2.5. Establishment of Standard Curves for the Acidity Measurement of Aqueous Solutions

The process for the establishment of pH standard curves based on measurement with fluorescence spectroscopy was as follows. A 10 μL volume of CdZnSeS-pATP suspension in DMSO (1.25 × 10^−2^ mg/mL) was added respectively to 1.5 mL of each of the aqueous solutions with a specific pH (1–7) as mentioned above, and then the fluorescence spectrum of the mixture was recorded immediately by Hitachi F-7000 fluorescence spectrometer (Hitachi, Tokyo, Japan). The standard curve was established by linear fitting of fluorescence intensity and pH values. In the measurement, the excitation wavelength was 365 nm, the scan speed was 1200 nm/min, and the photomultiplier tube (PMT) voltage was 700 V. The excitation slit and the emission slit were 20 nm and 10 nm, respectively.

When fluorescence spectroscopy was used to detect the acidity of aqueous solutions, the acidity values were obtained according to the fluorescence intensity in the standard curve. The detailed information about the aqueous solutions involved in this paper are respectively described in the captions of figures or tables.

### 2.6. Establishment of a Standard Curve for the Acidity Measurement of Paper

New filter paper (Whatman-Xinhua, Hangzhou, China) was soaked in aqueous solution with a specific pH (1–7) as mentioned above for 15 h with strict sealing, and then the soaked paper was taken out of the solution. The excess solution of the soaked paper was removed by adsorption with dry filter paper, and then the treated paper was placed in a vacuum drier for 30 min to make it dry more quickly. (To investigate the effect of CO_2_ on the acidity of paper, the treated paper was placed in a dryer containing CO_2_ for 1 d.) The dried paper was pressed at 10 MPa for 3 h by a tablet machine to make it flat. After that, the paper was cut into pieces of 35 mm × 25 mm, and then 5 μL of CdZnSeS-pATP suspension (4.40 × 10^−2^ mg/mL in chloroform) was dropped by pipette onto the center of the paper to ensure that the wet area was within the light spot of fluorescence measurement. The fluorescence spectra of the dried paper were recorded at the excitation wavelength of 365 nm. The standard curve for the acidity measurement of paper was established by linear fitting of the fluorescence intensity and pH. In the measurement, the excitation slit and the emission slit were 2.5 nm and 5 nm, respectively. Other conditions were the same as those in Section 2.5.

### 2.7. Paper Acidity Measured by Using Fluorescence Spectroscopy and a pH Meter

A 5 μL volume of CdZnSeS-pATP suspension (4.40 × 10^−2^ mg/mL in chloroform) was dropped by pipette onto the center of paper to ensure that the fluorescence area was contained in the light spot of fluorescence measurement. The fluorescence spectra of the dried papers were recorded by fluorescence spectrometer at the excitation wavelength of 365 nm, and the acidity values were obtained according to the fluorescence intensity in the standard curve. The conditions in the measurement of fluorescence spectroscopy are the same of those in Section 2.6. The detailed information about the paper samples involved in this analysis are respectively described in the captions of figures or tables.

For comparison, the traditional cold extraction method was also carried out to measure pH. A typical procedure is as follows. The papers, each with a different pH as mentioned above were, respectively, cut into pieces and the scraps were soaked in water with the ratio of 1 g:50 mL at RT for 1 h. The pH of each suspension was measured by pH meter. The pH was measured twice and the difference between two measurements could not exceed 0.2. The final pH is the average of two measurements [5].

## 3. Results and Discussion

### 3.1. Composition and Spectral Characteristics of CdZnSeS-pATP QDs

**Scheme 1:**
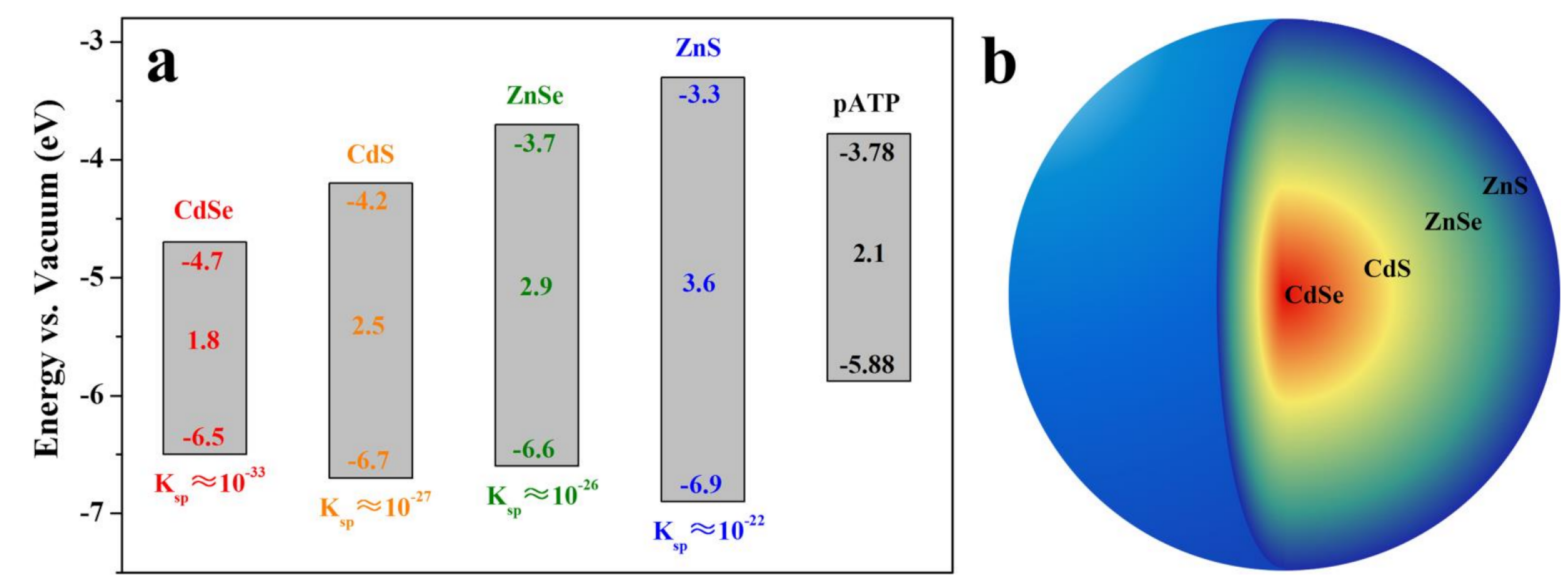
(**a**) Energy level diagrams of CdSe, CdS, ZnSe, ZnS and pATP. The energies of VB and CB, band gaps and Ksp are marked on the diagrams respectively [21,22]. (**b**) A schematic diagram of the chemical composition within a CdZnSeS QD.

The purchased CdZnSeS-OA QDs were synthesized by the single-step method where all the precursors of Cd, Zn, Se and S participated in the reaction in the same bath [19,20]. The four binary compounds made up by the four elements have different kinetic properties (see Scheme 1a for K_sp_ of four binary compounds), and the order of formation speeds of the four compounds (CdSe, CdS, ZnSe and ZnS) should be coincident with the sequence of radial distribution of the four compounds in the CdZnSeS QDs. Since the four compounds have similar chemical properties, well-defined interfaces between layers of different compounds are replaced by gradually varied alloy phases [21,22,23]. Scheme 1b shows the core–shell structure of CdZnSeS QDs with the chemical composition gradient, which treats CdSe and CdS as core, ZnSe as middle shell and ZnS as outer shell. It can be inferred that the band gap of CdZnSeS QDs increases with the radius by positions of valence bands (VB) and conduction bands (CB) of the four compounds (Scheme 1a), which proves the existence of a type Ⅰ structure [24].

**Figure 1:**
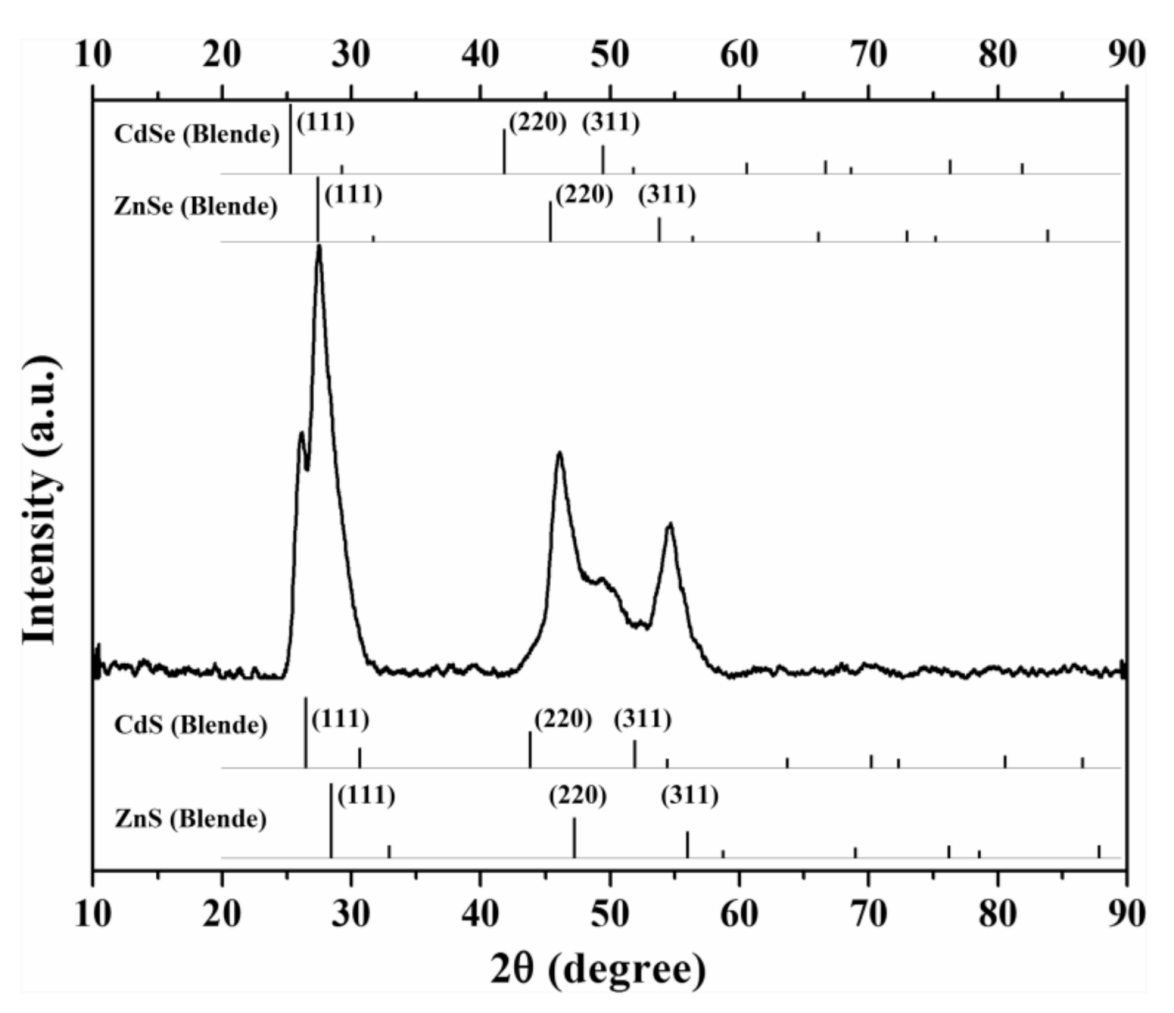
XRD patterns of CdZnSeS-pATP and the theoretical patterns of zinc-blende CdSe, ZnSe, CdS and ZnS.

To confirm the chemical composition of CdZnSeS-pATP QDs mentioned in Scheme 1, the powder XRD of CdZnSeS-pATP QDs was measured (Figure 1). In the pattern of XRD, CdZnSeS-pATP QDs exhibit the zinc blende structures of CdSe, CdS, ZnSe and ZnS. The three lines of ZnSe are closest to the three main peaks of CdZnSeS-pATP QDs, which may reflect that there is a thick ZnSe layer in a CdZnSeS-pATP QD.

**Figure 2:**
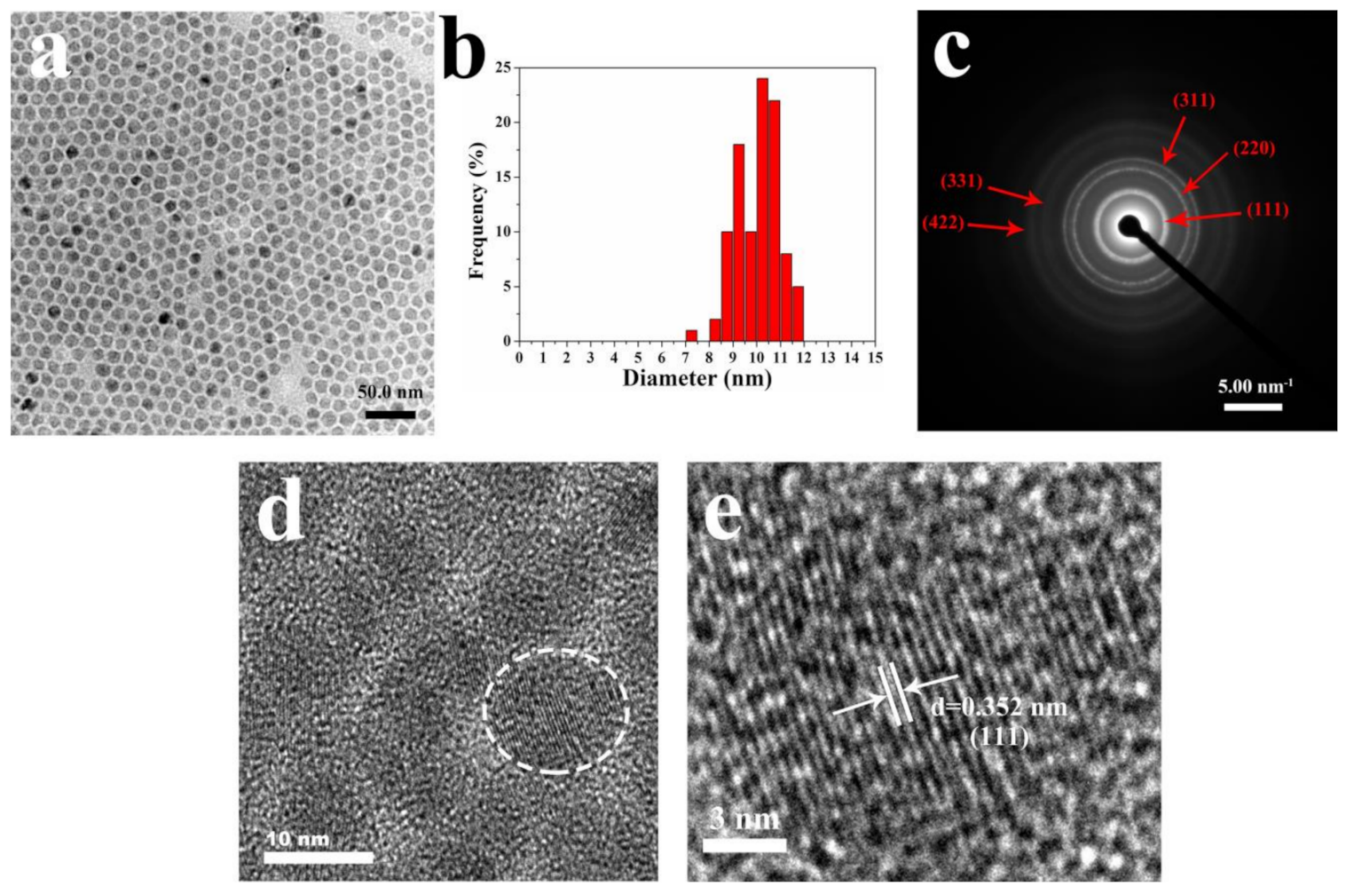
(**a**) TEM image of CdZnSeS-pATP QDs. (**b**) Size histogram with 100 particles in Figure 2a. (**c**) SAED pattern of particles in Figure 2a. The face symbols of diffraction rings of polycrystal are marked on the pattern. (**d**) HRTEM image of CdZnSeS-pATP QDs. The circled QD can be seen enlarged in (**e**) with its inter-planar spacing and face symbol indicated.

To further confirm the chemical composition and size distribution of CdZnSeS-pATP QDs, TEM was performed (Figure 2). Figure 2a shows the TEM image of CdZnSeS-pATP QDs. The nearly monodispersed nanocrystals are uniform in size, so that they can form well-ordered two-dimensional superlattices. The corresponding size distribution (Figure 2b) measured by analyzing 100 particles reveals that the mean diameter of QDs is 10.1 ± 8.8% nm. Obviously, the size distribution is narrow even though the particles have a multilayer alloy structure, and a narrow size distribution is the premise of a narrow emission linewidth. Diffraction rings of polycrystal in the SAED pattern of particles can be assigned to different lattice planes of the zinc-blende alloy phases of CdSe, CdS, ZnSe and ZnS (Figure 2c), which is consistent with XRD. The HRTEM image in Figure 2e shows well-resolved lattice fringes with a measured lattice spacing assigned to (111). These lattice fringes are continuous throughout the entire particle, revealing that the growth of gradient alloy shell appears to be epitaxial.

**Figure 3:**
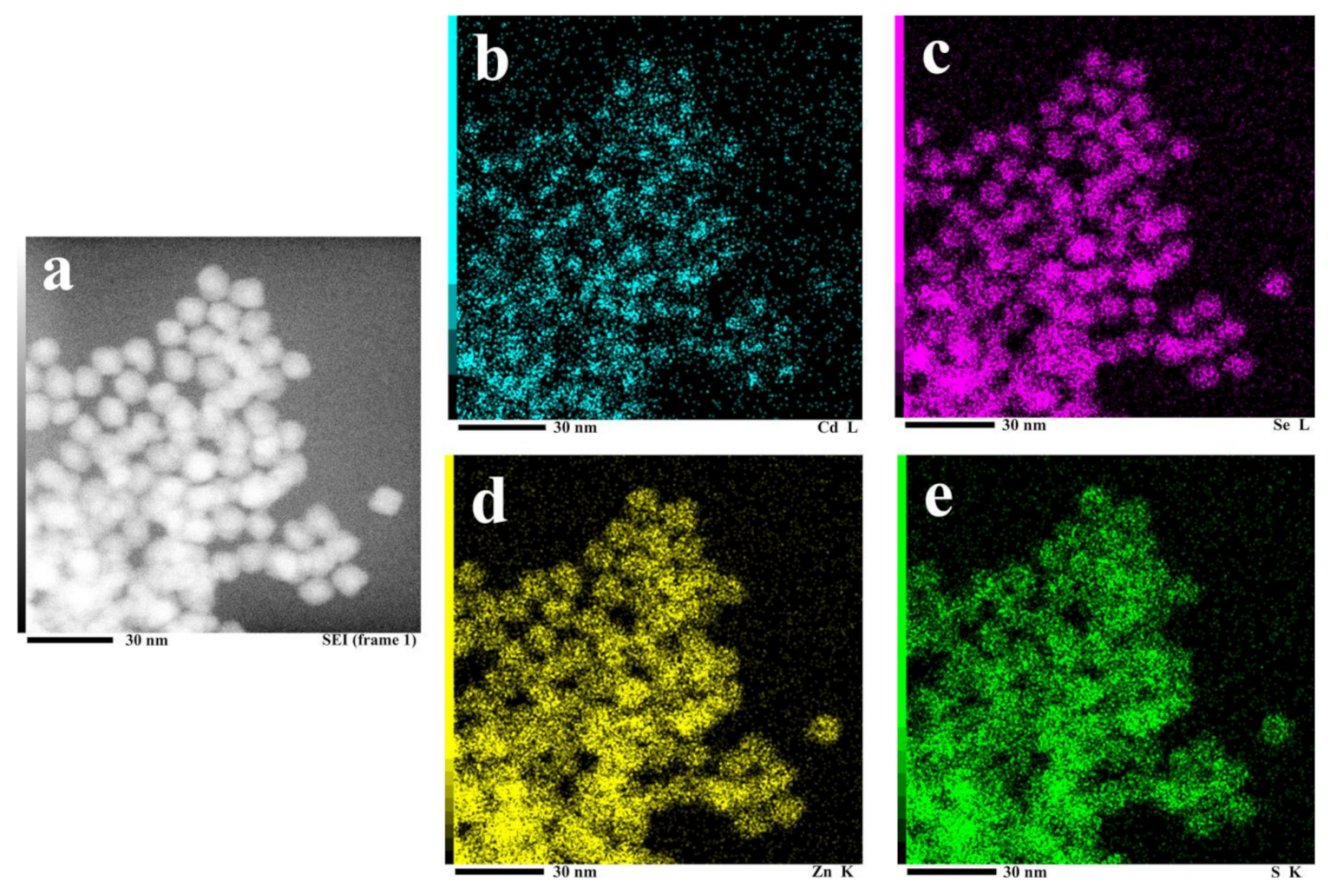
(**a**) The SEI image of CdZnSeS-pATP QDs. (**b**–**e**) EDS elemental mappings of Cd (**b**), Se (**c**), Zn (**d**) and S (**e**) corresponding to the CdZnSeS-pATP QDs array in Figure 3a.

EDS elemental mappings which can show the spatial distribution of elements were used to verify the hypothetical core–shell structure of CdZnSeS-pATP QDs (Figure 3). It clearly shows that the spatial distributions of Cd, Se, Zn and S are almost the same, implying that the prepared CdZnSeS-pATP QDs are composite materials. However, the brightness of the Cd located area is lower than that of other elements, and the brightness of the Zn located area is more similar to that of S than that of Se. These findings indicate that ZnS and ZnSe as shells, cover the core of CdSe and CdS in CdZnSeS. This characterization of the element distributions agrees with the proposed structure shown in Scheme 1b.

CdZnSeS-pATP QDs were prepared through OA in CdZnSeS-OA QDs exchanged by pATP. The surface ligand modification belongs to an X-type exchange in which both attacking and leaving ligands are negative-donors coordinating cationic-acceptors such as Zn atoms on surfaces of CdZnSeS QDs [25]. Apart from the fact that the bond energy of PhS-Zn in CdZnSeS-pATP is stronger than RCOO-Zn in CdZnSeS-OA [17], the removal of OA ligand by washing with the solvent ethanol also promotes the exchange [25]. Figure S1 shows the UV absorption spectra of the ethanol eluent of CdZnSeS-OA QDs after being treated by pATP. The UV absorption peak at 212 and 253 nm are assigned to the K band and B band of pATP respectively, and the two bands are derived from π–π* transition of the conjugated substituted benzene structure of pATP [26]. The UV absorption spectra indicate that both peaks at 212 and 253 nm gradually reduce with washing times of ethanol, and the absorbance spectrum for the seventh ethanol eluent is almost the same as that of ethanol [27], indicating that pATP physically absorbed on surfaces of CdZnSeS-pATP QDs could be removed through ethanol washing. The UV spectrum for the residue of the pATP treated CdZnSeS-OA after ethanol washing, CdZnSeS-OA and pATP, are shown in Figure S2. It clearly indicates that the UV spectra of the residue of the pATP treated CdZnSeS-OA after ethanol washing is very similar to that of pATP, implying that CdZnSeS-pATP QDs could be obtained through the ligand exchange method.

**Figure 4:**
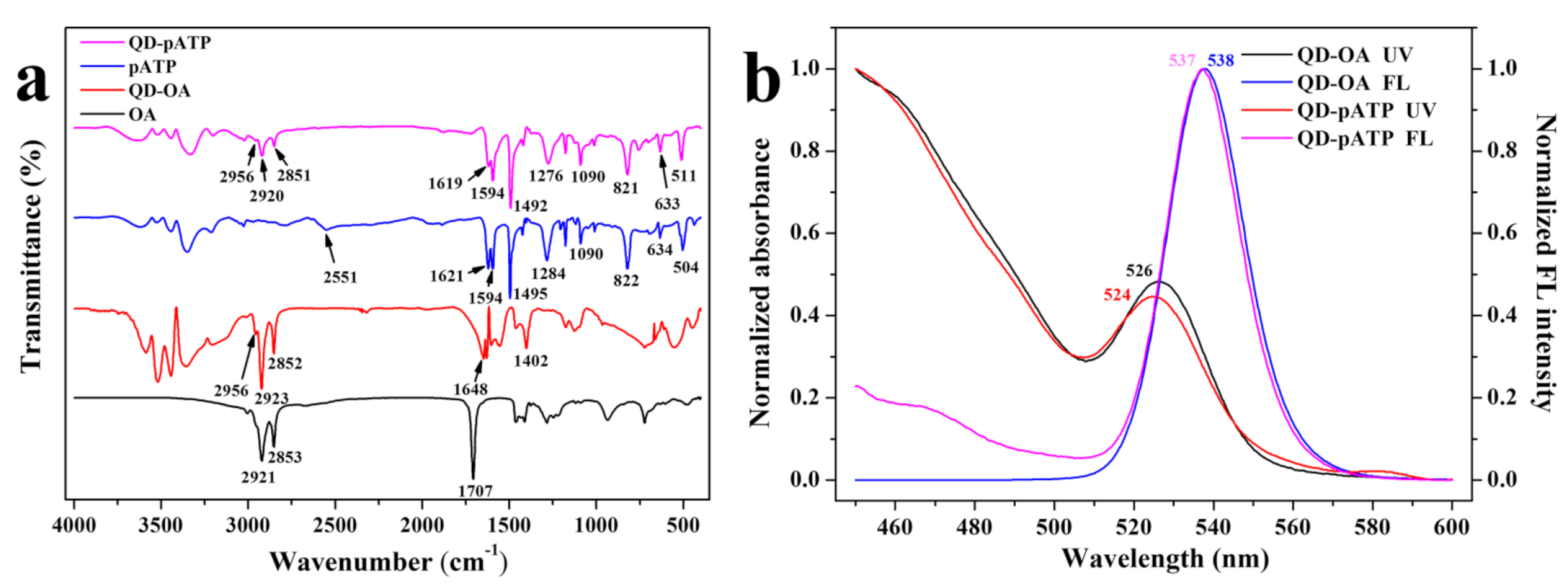
(**a**) FT-IR spectra of OA, CdZnSeS-OA QDs, pATP and CdZnSeS-pATP QDs. (**b**) Normalized UV-Vis spectra and fluorescence spectra of CdZnSeS-OA QDs in n-Hexane and CdZnSeS-pATP QDs in chloroform. (λex = 365 nm).

To further confirm ligand exchange of CdZnSeS QDs from OA to pATP, FT-IR spectra of OA, CdZnSeS-OA QDs, pATP and CdZnSeS-pATP QDs were measured and the results are shown in Figure 4a. For OA, there are characteristic peaks at 2921 and 2853 cm^−1^ attributed to the stretching vibration of C-H bonds in methylene and methyl, and the peak at 1707 cm^−1^ belongs to protonated carboxyl [17]. However, for CdZnSeS-OA QDs, except for the appearance of the peaks related to the stretching vibration of C-H bonds, the C=O stretching band at 1707 cm^−1^ disappears and new absorption bands appear at 1648 and 1402 cm^−1^ assigned to deprotonated carboxyl groups, revealing that the deprotonated carboxyl of OA binds on the surfaces of CdZnSeS QDs [28]. For CdZnSeS-pATP QDs, the signals at 1619, 1594 and 1492 cm^−1^ assigned to C=C stretching of aromatic rings, the signal at 1276 cm^−1^ assigned to C-N stretching, the bands at 1090 cm^−1^ attributed to S-Ar, and the characteristic peaks at 821, 633 and 511 cm^−1^ belonging to 1,4-substituted benzene, support the existence of pATP bound on the surfaces of CdZnSeS-pATP QDs. Additionally, the loss of 2551 cm^−1^ assigned to S-H of free pATP suggests the formation of S-M bonds in CdZnSeS-pATP QDs [16]. The very weak residual signals at 2956, 2920, 2851 cm^−1^ in the spectra of CdZnSeS-pATP QDs imply a very small amount of OA in CdZnSeS-pATP QDs.

Figure 4b shows the UV-Vis spectrum and the fluorescence spectrum of CdZnSeS-OA QDs and CdZnSeS-pATP QDs. The absorption peaks of CdZnSeS-OA QDs and CdZnSeS-pATP QDs are 526 nm and 524 nm, respectively, and the emission peaks of the corresponding samples are 538 nm and 537 nm, respectively. For CdZnSeS-OA QDs and CdZnSeS-pATP QDs, the absorption and the respective emission peaks are almost same, indicating that the UV-Vis absorbance and fluorescence originate from CdZnSeS QDs. Additionally, the narrow FWHM (ca. 22 nm) of fluorescence emission peaks shows the excellent uniformity of size and chemical construction of CdZnSeS-pATP QDs. In summary, the characteristics of CdZnSeS-pATP QDs have confirmed the core–shell structure with a chemical composition gradient of CdZnSeS-pATP QDs and the successful ligand exchange of QDs from OA to pATP.

### 3.2. Fluorescence Response of CdZnSeS-pATP QDs to Acidity

**Figure 5:**
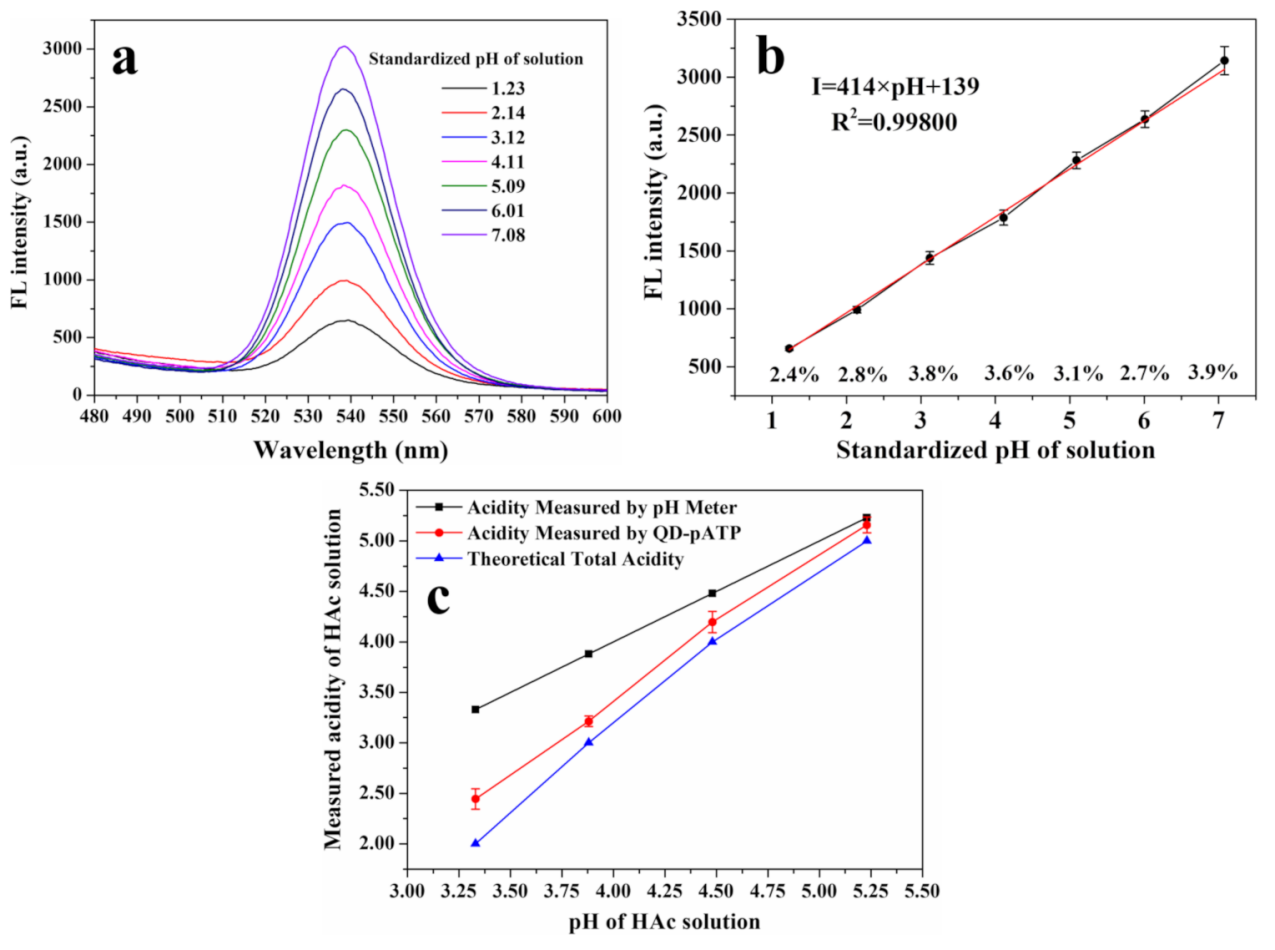
(**a**) The response of the fluorescence spectra of CdZnSeS-pATP QDs in aqueous solutions with standardized pH values. (**b**) The linear dependency between the fluorescence intensity at maximum emission 537 nm and pH. The error bars represent the standard deviation of four measurements. The RSD of four measurements were marked at each pH. The red line represents the fitting curve of measured values. (**c**) The measured acidity of HAc solutions of 1.0 × 10^−2^ M, 1.0 × 10^−3^ M, 1.0 × 10^−4^ M and 1.0 × 10^−5^ M by pH meter and CdZnSeS-pATP QDs. The error bars represent the standard deviation of four measurements. (C_QDs_ = 8.28 × 10^−5^ mg/mL, Water:DMSO = 150:1(*V*/*V*), λex = 365 nm).

To evaluate the fluorescence response behaviors of CdZnSeS-pATP QDs to the acidity (including free protons and undissociated protons) of aqueous solutions, the change in the fluorescence spectrum of CdZnSeS-pATP QDs with the variation of aqueous solutions was measured (Figure 5). It is observed that the fluorescence intensity at maximum emission peak 537 nm of CdZnSeS-pATP QDs increases with pH values ranging from 1 to 7. A good linearity can be found between the fluorescence intensity and pH with the standard fitting formula I = 414 × pH + 139 (R^2^ = 0.99800). (Figure 5b) Acceptable system suitability and method precision were confirmed with a relative standard deviation (RSD) of less than 4%, and the corresponding results are shown in Figure 5b. In order to confirm the effectiveness of this method in the determination of total acidity (free protons and bound protons), the measured acidity of acetic acid (HAc) aqueous solution by pH meter and CdZnSeS-pATP QDs is shown in Figure 5c. The corresponding acidity values of HAc solutions detected by two methods are shown in Table S1 and the corresponding fluorescence spectra of HAc solutions are shown in Figure S3. The results indicated that the acidity measured by CdZnSeS-pATP QDs approaches the theoretical total acidity of HAc. As expected, the acidity of HAc determined by pH meter differs greatly from the theoretical acidity because it only responds to free protons. However, CdZnSeS-pATP QDs respond not only to free protons but also to undissociated protons in aqueous solution, and so the corresponding pH values determined by CdZnSeS-pATP QDs are obviously lower than those measured by pH meter. In conclusion, CdZnSeS-pATP QDs as sensors, exhibit an excellent optical response to the total acidity of aqueous solutions.

**Figure 6:**
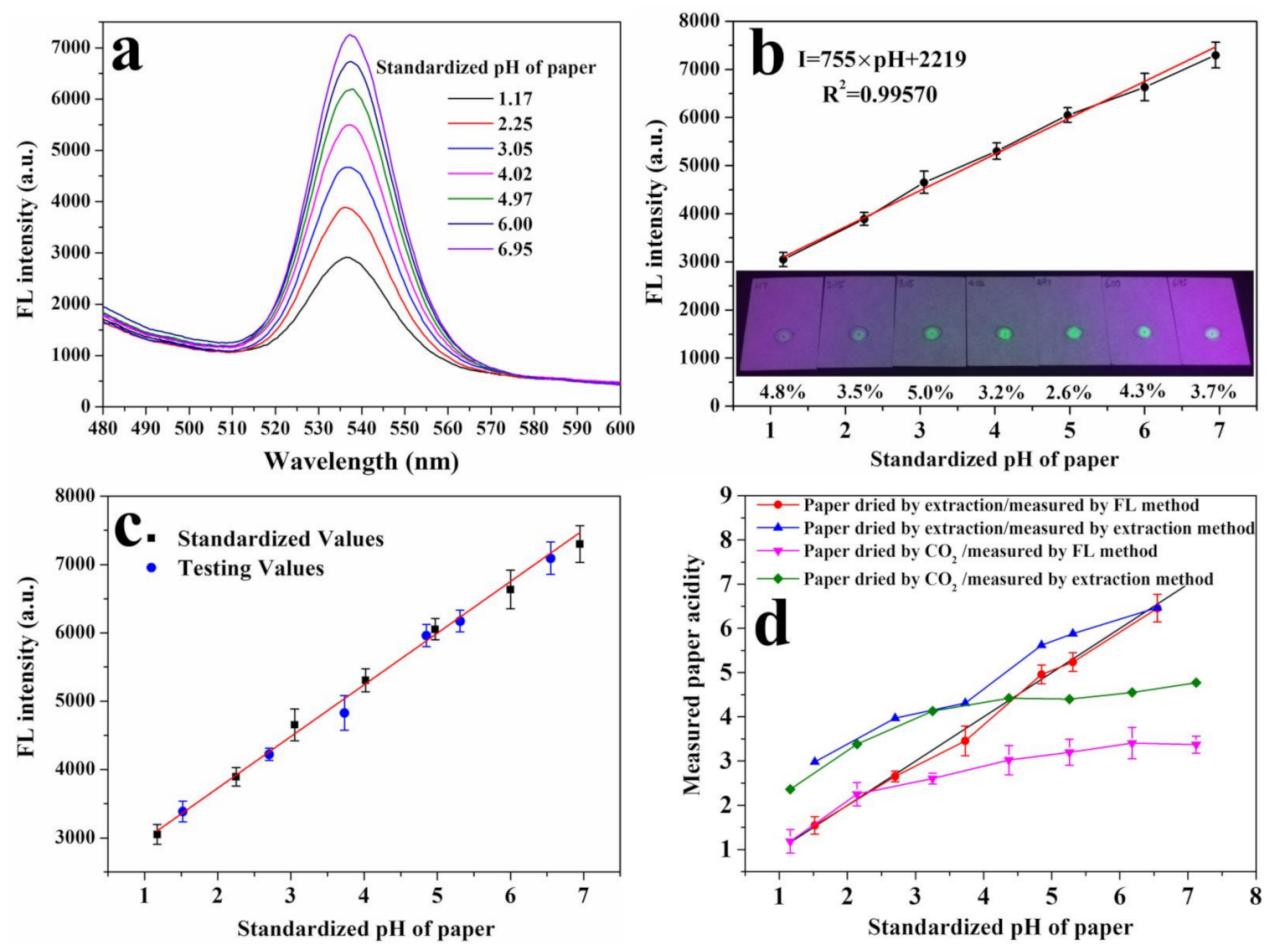
(**a**) The fluorescence spectra of CdZnSeS-pATP QDs on the paper with different standardized pH values. (**b**) The linear dependency between the fluorescence intensity at maximum emission 537 nm and pH. The error bars represent the standard deviation of four measurements. The RSD of four measurements were marked at each pH. Inset shows the fluorescence photographs of the paper with different standardized pH values. The circles marked on the paper indicate the area of the light spot of fluorescence measurement. The red line represents the fitting curve of measured values. (**c**) The plot of fluorescence intensity of testing values at the maximum emission wavelength 537 nm as a function of pH. The error bars represent the standard deviations of four measurements. (**d**) The measured acidity of the paper with different standardized pH values as determined by different methods. The error bars represent the standard deviations of four measurements. (λex = 365 nm).

Generally, cellulose in paper can be degraded to form various organic acids [29], and the free protons can be adsorbed onto cellulose [7]. Acid catalysis is an important factor in the degradation of paper cellulose [30]. Therefore, acid-catalyzed cellulose degradation in paper has been of wide concern. It should be noted that the determination of paper acidity is most commonly made by the determination of free protons by pH meter. In fact, free protons don’t fully represent paper acidity. Adsorbed protons and undissociated weak acids also play an important role in the degradation of paper fibers [7]. For determination of the total acidity of paper, the paper samples with standardized pH values were prepared by soaking a filter paper in a standardized pH aqueous solution according to Moorthy’s work [8]. The acidity of the prepared paper with standardized pH was determined by CdZnSeS-pATP QDs and pH meter, respectively. The results are shown in Figure 6. The fluorescence intensity linearly increases with pH, and the standard fitting formula for four measurements is I = 755 × pH + 2219 (R^2^ = 0.99570). The results shown in Figure 6b indicate that CdZnSeS-pATP QDs have an excellent optical response to the acidity of paper.

In order to verify the availability of the standard curve, the fluorescence intensities of CdZnSeS-pATP QDs on the paper after soaking in the specific pH aqueous solutions were determined (Figure 6c). It is clear that the testing dots fit well with the standard curve. This result demonstrates that paper acidity could be determined by the proposed fluorescence method. To explore differences in the paper acidity measured by the traditional and the fluorescence method, the acidity values of the pH calibrated paper were measured by the two methods respectively, and the results are shown in Figure 6d. For the fluorescence method, the determined pH is very similar to the calibrated pH. However, the measured pH by the pH meter is higher than the calibrated pH, and the difference increases especially with the decrease of pH. As conjectured, the reason for this may be that protons are adsorbed by the paper cellulose in acidic conditions.

In order to explore the effect of CO_2_ on the pH measurement, the humid paper with the standardized pH was dried in a CO_2_ atmosphere, and pH values of the corresponding dried samples were determined by the pH meter and the fluorescence method, respectively. The results are shown in Figure 6d. The corresponding measured acidity values of paper in Figure 6d are shown in Table S2. The corresponding fluorescence spectra of filter paper dried by extraction and CO_2_ are shown in Figure S4a,b, respectively. For both the traditional and the fluorescent methods, the measured acidity of the paper dried in a CO_2_ atmosphere was higher than that of paper dried by extraction, especially in the higher pH range. Obviously, this result is attributed to the neutralization by the acidic gas CO_2_ [31,32]. Additionally, the measured acidity of the paper dried in a CO_2_ atmosphere, as determined by the fluorescence method, is higher than by the traditional method. This difference is related to the pH meter which only responds to free protons as mentioned above. Evidently, the harm of acid gases to paper should not be neglected [33]. In summary, CdZnSeS-pATP as sensors exhibit excellent optical responses to the total acidity of both aqueous solutions and paper.

### 3.3. Mechanism of the Fluorescence Response of CdZnSeS-pATP QDs to Acidity

**Scheme 2:**
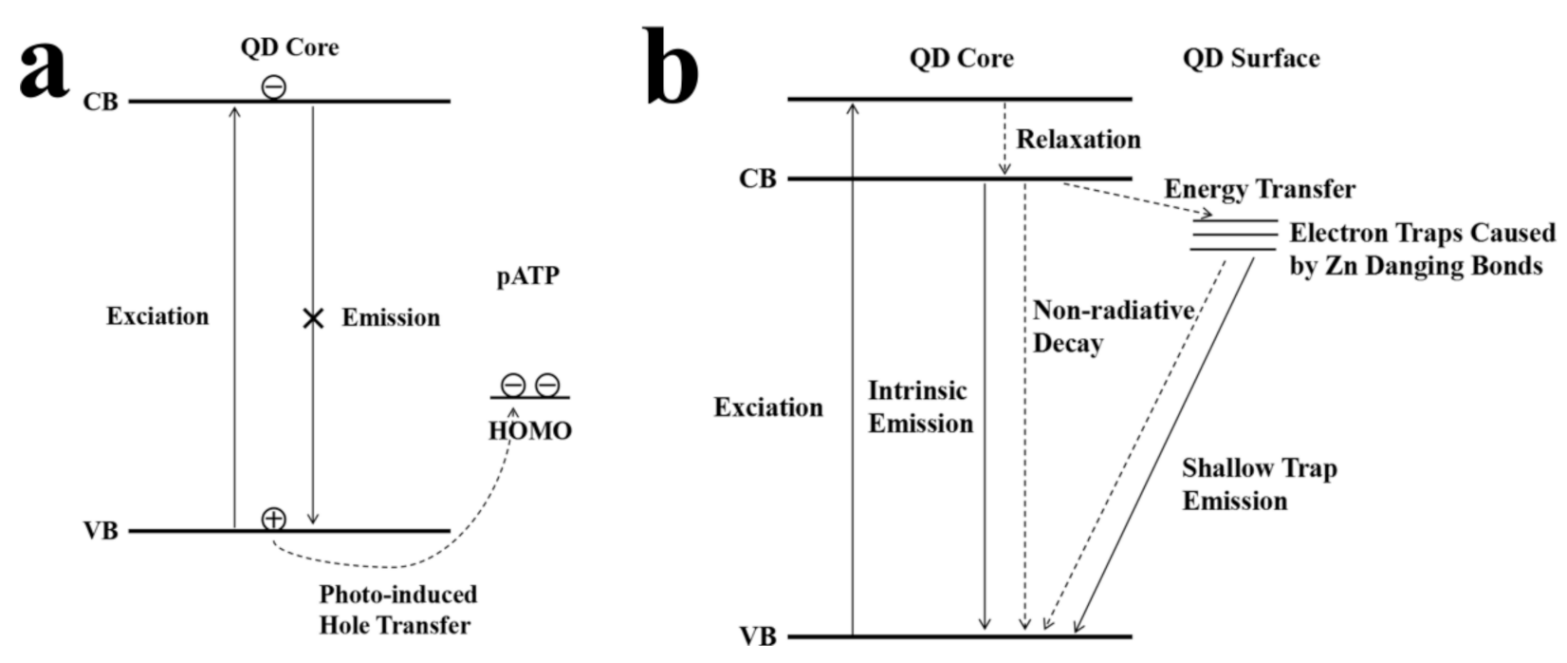
The schematic diagrams of (**a**) the mechanism on the quenching of CdZnSeS QDs fluorescence by pATP and (**b**) the mechanism on the dual-channel fluorescence emission of CdZnSeS-pATP QDs.

To understand the nature of the fluorescence response of CdZnSeS-pATP QDs to acidity, the mechanism of fluorescence response was investigated. The fluorescence response behaviors of CdZnSeS-OA and CdZnSeS-pATP were detected, respectively (Figure S5). It was found that the fluorescence of CdZnSeS-pATP QDs could respond to pH rather than CdZnSeS-OA QDs. It can be inferred that the mechanism of fluorescence response is determined by the relationship between acidity and the interaction of CdZnSeS QDs and pATP ligands. Table 1 indicates that the fluorescence quantum yield of CdZnSeS-pATP QDs is markedly lower than that of CdZnSeS-OA QDs, and the fluorescence quantum yield of CdZnSeS-pATP QDs decreases with increased acidity. It is generally thought that the thiol ligand could quench fluorescence of QDs due to the transfer of holes from QDs to the thiol groups [34,35]. An electron–hole pair exciton forms when a photon is absorbed by a QD. If the VB of the QD is located at a lower energy level than the HOMO of the thiol ligand, the transfer of the hole from the QD to the thiol is energetically favorable [36]. As the thiol ligand, the phenyl group of a pATP molecule can disperse the positive charge, which makes the hole stable [37]. As a result, radiative recombination of the exciton is not possible, and QDs keep a lower fluorescence quantum yield [38,39]. Since the four binary compounds in CdZnSeS QDs have almost the same VB energy (difference < 0.4 eV in Scheme 1a), CdZnSeS QDs have nearly constant energy of VB as a function of radius, which contributes to the movement of holes from cores to surfaces of QDs. For pATP, its HOMO is −5.88 eV, and the valence band edge of CdZnSeS QD is expected to be around −6.5 eV (Scheme 1a). Therefore, pATP can effectively capture the photo-generated holes of CdZnSeS QDs, and CdZnSeS-pATP QDs have a weaker fluorescence. The mechanism on the fluorescence of CdZnSeS QDs quenched by pATP is showed in Scheme 2a.

To better understand the fluorescence dynamics, the time-resolved fluorescence spectra of CdZnSeS-OA QDs and CdZnSeS-pATP QDs were determined using a 340 nm laser pulse as the excitation source (Figure S6). Both the emission intensities of CdZnSeS-OA QDs and CdZnSeS-pATP QDs recorded at 537 nm are fitted best to biexponential functions. For the OA ligand, CdZnSeS-OA QDs exhibit lifetimes of 13.92 ns (ƒ_1_ = 0.971) and 46.28 ns (ƒ_2_ = 0.029), respectively (Table 1). The short-lived emission at the band edge wavelength is attributed to the intrinsic channel, and the long-lived emission derives from the shallow trap caused by Zn dangling bonds on CdZnSeS QD surfaces in which the energy is close to that of the CB of QDs [40,41] (Scheme 2b). Due to few trap states existing on the freshly synthesized CdZnSeS-OA QDs, their fluorescence properties are dominated by intrinsic emission (97.1%). For the pATP ligand, the CdZnSeS-pATP QDs exhibit lifetimes of 5.31 ns (ƒ_1_ = 0.591) and 11.70 ns (ƒ_2_ = 0.409), respectively. A higher proportion of trap emission than CdZnSeS-OA QDs may be because the larger exposed surface caused by stronger steric hindrance of the phenyl of pATP, is beneficial to the formation of Zn dangling bonds. Additionally, both the fluorescence lifetimes of dual-channel emission of CdZnSeS QDs decrease after ligand modification because the larger exposed area has promoted the nonradiative transition rate constants of both the intrinsic and trap emissions, due to the leakage of exciton wave functions [42].

**Scheme 3:**
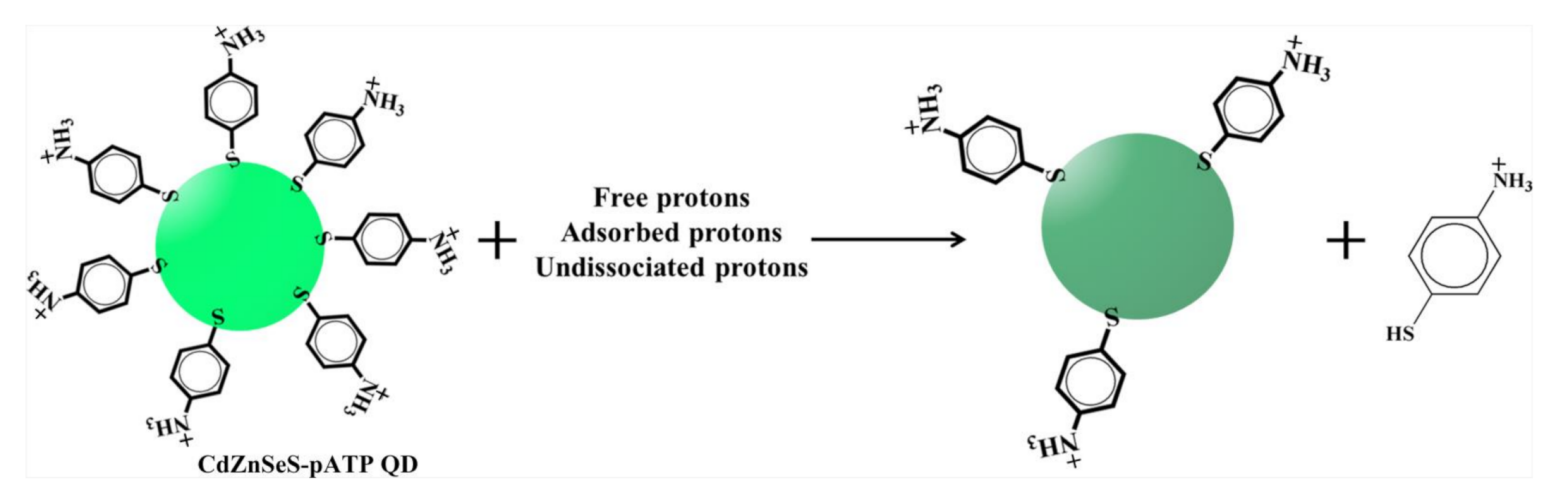
The schematic diagram of the mechanism of fluorescence response of CdZnSeS-pATP QDs to paper acidity.

The mechanism of the fluorescence response of CdZnSeS-pATP QDs to acidity is inferred as follows. It is reported that the low fluorescence intensity under acidic conditions results from the dissociation of the thiol ligand on QDs due to protonation of the surface-binding thiolate [43,44,45]. As the thiol ligand, pATP (pK_a_ = 6.86) [46] can also dissociate from CdZnSeS QDs, which causes the direct exposure of CdZnSeS QD surfaces to solvent molecules. The contact between the nanocrystal surfaces and surroundings leads to a higher nonradiative relaxation rate, resulting in the reduced fluorescence quantum yield and lifetime at lower pH values [42] (Table 1). The decreasing proportion of band gap emission and increasing percentage of trap emission with decreasing pH, implies that the more the dissociation of pATP ligands, the greater the surface of CdZnSeS QDs is exposed to solution. Scheme 3 shows the mechanism of the fluorescence response of CdZnSeS-pATP QDs to paper acidity.

In order to verify the dissociation of pATP from CdZnSeS-pATP QDs under acidic conditions, ^1^H NMR spectra of CdZnSeS-pATP QDs supernate in a D_2_O/DMSO-d6 mixture at pH values of 6 and 1 were detected respectively (Figure S7). The pH of the mixtures was adjusted with deuterium hydrochloric acid (DCl), and tetramethylammonium chloride (TMAC), with same concentrations used as internal standard substances in the suspensions of CdZnSeS-pATP QDs with different pH values. After centrifugation, CdZnSeS-pATP QDs were deposited but TMAC and the dissociated pATP ligands were still in the supernatant. At the pH of 6, the resonance at 3.05 ppm is attributed to TMAC. Free pATP exhibited two quasi-doublets centered at 6.61 and 7.17 ppm, corresponding to the aromatic protons [47,48]. The molar ratio of free pATP to TMAC was 0.12:1, calculated from the relative integral intensity of the peaks. With the pH adjusted to 1, the molar ratio of free pATP to TMAC increased to 3.53:1 (ca. 30-fold), indicating that more pATP ligand dissociated from CdZnSeS-pATP QDs under acidic conditions.

In particular, we propose that the mechanism of the quenching of CdZnSeS-pATP QDs fluorescence by the dissociation of pATP is different from that reported in the literature [16]. The explanation of the mechanism reported in the literature is as follows. The HOMO of pATP ligand locates at a relatively high energy position above the VB of QDs, and so pATP could serve as the trapper to accept photo-generated holes of QDs in neutral conditions. The inhibition of the native electron–hole recombination of QDs results in the quenching of fluorescence of the QDs. In acidic conditions, the amino groups of pATP on QDs can easily react with protons to form ammonium ions, which reverses the relative energy positions of the HOMO of pATP, and VB of QDs. The new resulting energy levels are no longer suitable for the hole transfer and so fluorescence of the QDs is switched on [16]. However, in our experiments, the fluorescence of CdZnSeS-pATP decreases with increases in acidity. The reason for the discrepancy between our findings and the reports in the literature may be because of the difference in structure of QDs. In the literature, the QDs have the CdSe/ZnS core–shell structure, and the surface composition of CdSe/ZnS is a nearly pure ZnS phase. However, our QDs are quaternary alloy QDs CdZnSeS, and the surface composition of CdZnSeS is the alloy phase of ZnS and ZnSe. Since the Pauling electronegativity of Se (2.4) is lower than that of S (2.5), the positive charge quantity of Zn atoms close to Se is smaller than that near S, which causes the bond energy of PhS-ZnSe to be weaker than that of PhS-ZnS [49]. It can be inferred that pATP easily leaves from surfaces of CdZnSeS QDs which contain Se due to the protonation of pATP [50].

### 3.4. The Influences of Impurities in Paper on the Fluorescence of CdZnSeS-pATP QDs

**Figure 7:**
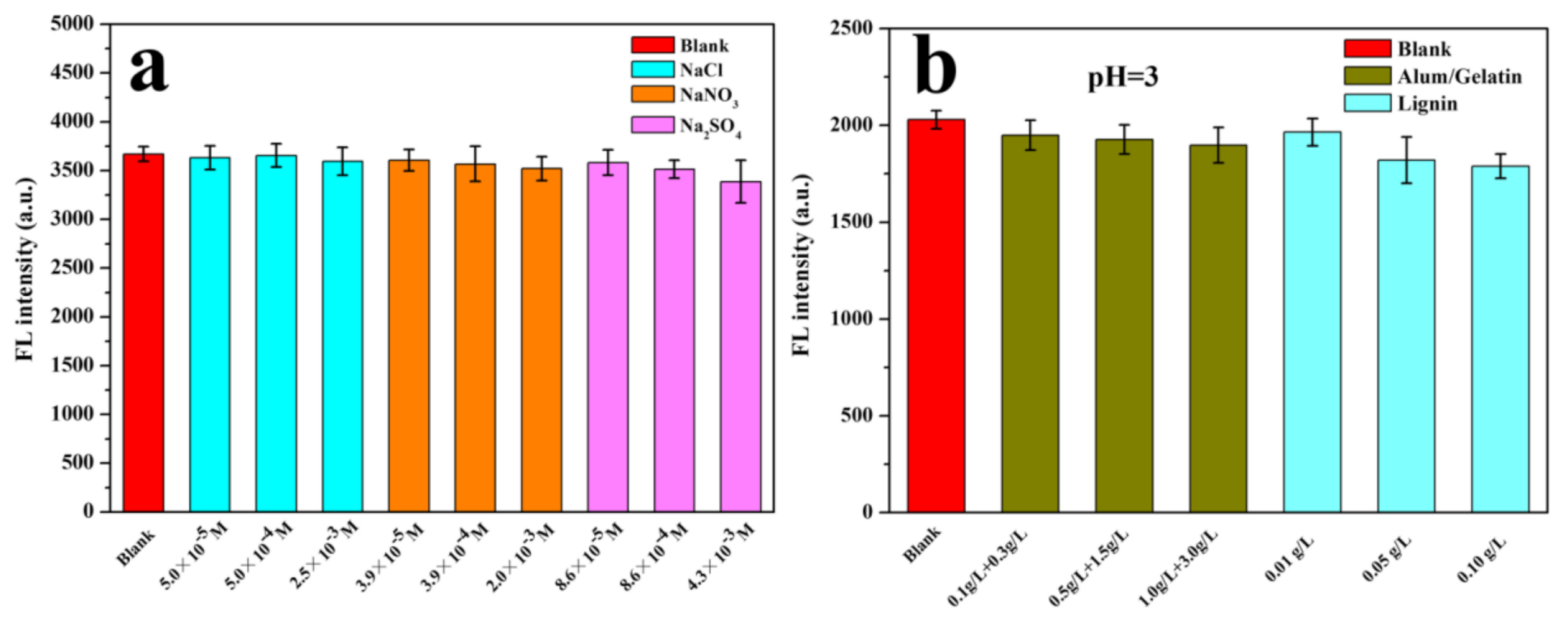
(**a**) The fluorescence intensities of CdZnSeS-pATP QDs at 537 nm in presence of NaCl, NaNO_3_ and Na_2_SO_4_ at different concentrations. The error bars represent the standard deviations of four measurements. (**b**) The fluorescence intensities of CdZnSeS-pATP QDs at 537 nm in presence of gelatin-alum and lignin at different concentrations, at a pH of 3. The error bars represent the standard deviations of four measurements. (C_QDs_ = 1.15 × 10^−4^ mg/mL, Water:DMSO = 150:1(*V*/*V*), λex = 365 nm).

To verify the selectivity of the fluorescence method using CdZnSeS-pATP QDs to detect acidity, the fluorescence behaviors of CdZnSeS-pATP QDs were measured in the presence of impurities. The impurities in paper, especially the components which have considerable content and a different chemical structure to cellulose, may quench fluorescence of CdZnSeS-pATP QDs. The effects of some impurities including inorganic salts, gelatin-alum solution and lignin on the fluorescence of CdZnSeS-pATP QDs were investigated. In considering the fact that the concentrations of Cl^-^, NO^3-^ and SO_4_^2-^ aqueous solutions were decided by their mass fraction in old newspaper, as measured by the hot water extraction method [33], Figure 7a shows the fluorescence intensities of CdZnSeS-pATP QDs in the presence of NaCl, NaNO_3_ and Na_2_SO_4_ at different concentrations. The corresponding fluorescence spectra are shown in Figure S8a. We can conclude that Cl^-^, NO^3-^ and SO_4_^2-^ have no significant effects on the fluorescence intensity of CdZnSeS-pATP. Gelatin-alum solution is often used for sizing paper [51], and lignin is considerable in many papermaking raw materials, such as the phloem fibers of paper-mulberry (ca.14 wt%), whinghackberry (ca. 10 wt%) and white mulberry (ca. 8 wt%) and the stem fibers of bamboo (ca. 30 wt%) and straw (ca. 14 wt%) [52]. Therefore, the effects of gelatin-alum and lignin on the fluorescence of CdZnSeS-pATP QDs were also investigated. Figure 7b shows the fluorescence intensities of CdZnSeS-pATP QDs in the presence of gelatin-alum and lignin at different concentrations. The corresponding fluorescence spectra of gelatin-alum and lignin are shown in Figure S8b,c, respectively. Since both gelatin-alum and lignin aqueous solutions are weakly acidic, the determined solutions were adjusted to a pH of 3 by hydrochloric acid to eliminate the difference in acidity. Except for the slight decrease of the fluorescence intensity with an increase of lignin content, because of a small amount of absorption of 365 nm excitation light by lignin (Figure S9), the fluorescence intensity is stable with the changes of gelatin-alum content. In short, the influences of typical impurities in paper-based relics on the fluorescence of CdZnSeS-pATP QDs can be considered negligible, and CdZnSeS-pATP QDs as potential pH probes show a high selectivity to protons in paper.

### 3.5. Detection of the Total Acidity of Paper-based Relics

**Figure 8:**
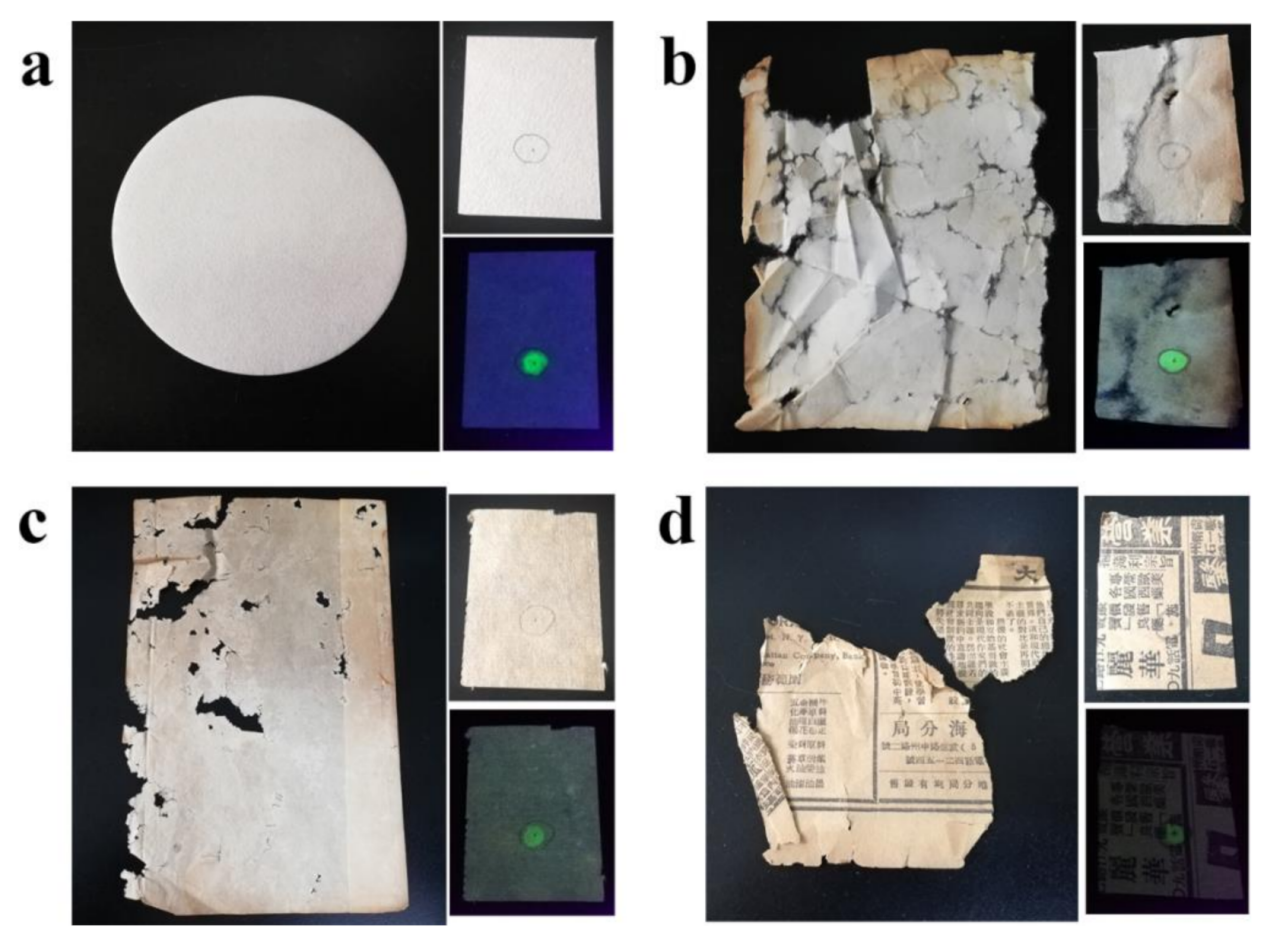
The photographs of four paper samples. (**a**) Filter paper. (**b**) Ancient book title page of Mianlian paper from the 19th century. (**c**) Ancient book title page of bamboo paper from the 19th century. (**d**) Newspaper:“China Times, Xuedeng” from 1922. Upper-right and lower-right corners show the photographs of paper samples containing CdZnSeS-pATP QDs under daylight and 365 nm UV light, respectively.

Due to the high selectivity of CdZnSeS-pATP QDs to protons in paper, the standard curve of the fluorescence response of CdZnSeS-pATP QDs to paper acidity is expected to be applicable for the measurement of acidity of true paper-based relics. Three kinds of typical paper-based relics were chosen to detect the total acidity: Mianlian paper from the 19th century, bamboo paper from the 19th century and newspaper from 1922. The filter paper which is nearly pure cellulose was also detected for comparison (Figure 8a). Mianlian paper belonging to Xuan paper contains 30~40 wt% phloem fibers from whinghackberry and 60~70 wt% stem fibers from straw in a sand paddy [53]. Due to a lesser lignin content in the raw materials, and storage for one month after soaking the raw materials in a lime slurry and a subsequent boiling process, the lignin content in Mianlian paper is very low and the whiteness of the paper is good [54]. The yellow edge of the paper was caused by the contact with light and oxygen (Figure 8b). While bamboo raw material underwent two alkali boiling processes to remove lignin [55], the high lignin content of bamboo makes the paper look light yellow because of the oxidation of lignin [56] (Figure 8c). Old newspapers were usually made of Groundwood pulp which hardly dissolves out the lignin in the raw materials, and the lignin content of the newspaper is the highest among the four paper samples and its yellow color is the darkest (Figure 8d). Figure S10a shows the normalized UV-Vis absorption spectra of four paper samples. The UV absorption peaks around 230 nm are assigned to cellulose, and the maximum absorption around 270 nm originates from nonconjugated phenolic groups in lignin [57]. Since absorbances of cellulose are normalized, the height of the 270 nm peak can reflect the lignin content in paper samples. The sequence of lignin content acquired by absorption spectra is consistent with that recorded in the literature. Figure S10a also shows that the four paper samples have little absorption of 365 nm excitation light, which is beneficial to the accuracy and reproducibility of the fluorescence method. Furthermore, the photographs of the four paper samples containing CdZnSeS-pATP QDs under daylight (upper-right corners in Figure 8) show fluorescent probes of QDs have no effect on the appearance of paper-based relics.

The acidity of the four paper samples was determined by the fluorescence method (Figure S10b) based on the standard fitting formula mentioned above. The pH values of the paper measured by the fluorescence or by the cold extraction method (Table 2) show the order of acidity of the four paper samples to be; Newspaper (the most acidic), bamboo paper, Mianlian paper and filter paper, which is consistent with the sequence of lignin content. Lignin may have an important influence on the acidity of paper because lignin is a kind of weak acid and it can be degraded into micromolecular organic acid by oxidation over a long time [56]. The important phenomenon is that acidity of paper measured by the fluorescence method is obviously higher than that measured by the cold extraction method. We believe that the cold extraction method can detect only free protons and the fluorescence method can detect total acidity, and the difference in acidity should be attributed to the bound protons of the phenolic hydroxyl of lignin and carboxyl of organic acids formed by the degradation of lignin. The low total acidity in Mianlian paper can also be owed to the wrinkles of phloem fibers of whinghackberry which can store CaCO_3_ particles rooting from the residual lime from the alkali treatment [58]. The total acidity of the older Mianlian paper and bamboo paper is lower than the younger newspaper, which shows the superiority of Chinese ancient handmade paper. In short, the fluorescence method based on CdZnSeS-pATP QDs can detect the total acidity of paper-based relics which reflects the real degree of acidification of the paper, but traditional cold extraction methods cannot.

## 4. Conclusions

Quaternary alloy CdZnSeS-pATP QDs were prepared, and their fluorescence behaviors were investigated. Their use in a fluorescence-based method for acidity measurement of paper-based relics was also investigated. The CdZnSeS-pATP QDs with a mean diameter of 10.1 ± 8.8% nm are nearly monodispersed and have narrow FWHM of fluorescence emission peaks. The following conclusions could be drawn:

- The prepared CdZnSeS-pATP QDs have a CdSe and CdS core, ZnSe middle shell, and ZnS outer shell. The alloy structure of CdZnSeS QDs could eliminate lattice mismatch to acquire high fluorescence quantum efficiency (88.9% for CdZnSeS-OA).
- OA ligand could be exchanged by pATP at the surface of CdZnSeS QDs to obtain specific optical properties concerning a response to acidity because of the dissociation of the pATP from the QDs under acidic conditions. The fluorescence response of CdZnSeS-pATP QDs to the acidity of paper samples has a wide acidity sensing range (1–7), high sensitivity (ca. 2.5-fold fluorescence intensity enhancement with pH values increasing from 1 to 7), good linearity (R^2^ = 0.99570) and excellent reproducibility (RSD of four measurements less than 5% at the same pH).
- A set of standard operations was implemented to determine paper acidity by using the fluorescence method, which can ensure excellent reproducibility. Additionally, the effects of some impurities, such as inorganic salts, gelatin-alum and lignin, on the fluorescence of CdZnSeS-pATP QDs are negligible.
- The acidity measured by the fluorescence method is higher than that by the cold extraction method in paper-based relics, which proves the conjecture that the cold extraction method detects only free protons, while the fluorescence method can detect the total acidity including free protons, adsorbed protons and undissociated protons in paper-based samples.

In addition, CdZnSeS-pATP QDs could be used as fluorescence probes to detect the total acidity of paper-based relics in a nondestructive way by using optical fibers [59]. To the best of our knowledge, this is a nondestructive fluorescence assay based on QDs to measure the total acidity of paper-based relics for the first time.

## Figures and Tables

**Table 1:** The quantum yield and biexponential fit parameters from fluorescence lifetime measurement ^1^.

Sample	Solvent	Quantum Yield	τ_1_(ƒ_1_)	τ_2_(ƒ_2_)	τ_avg_ (ns)	R-Square
QD-OA	Chloroform	0.889	13.92(0.971)	46.28(0.029)	16.84	0.99715
QD-pATP	Chloroform	0.340	5.31(0.591)	11.70(0.409)	9.17	0.99799
QD-pATP(pH = 1.31)	DMSO/Water(1:1)	0.041	1.73(0.359)	6.40(0.641)	5.79	0.99532
QD-pATP(pH = 4.11)	DMSO/Water(1:1)	0.278	3.93(0.485)	8.19(0.515)	6.87	0.99639
QD-pATP(pH = 6.98)	DMSO/Water(1:1)	0.295	4.20(0.554)	8.81(0.446)	7.10	0.99279

^1^ The fluorescence decay was analyzed using the expression F(t) = a_1_exp(−t/τ_1_) + a_2_exp(−t/τ_2_), where τ_1_ and τ_2_ were the lifetimes. The values in parentheses indicate the fraction of the corresponding lifetime component. The average lifetime values were calculated using the expression τ_avg_ = ∑a_i_τ_i_^2^/∑a_i_τ_i_.

**Table 2:** The acidity of paper-based relics detected by two methods.

Sample	Paper Acidity Measured by FL Method ^1^	Paper Acidity Measured by Cold Extraction Method	Difference
Filter paper	6.50 ± 0.19	6.72	0.22
Mianlian paper	5.33 ± 0.31	5.75	0.42
Bamboo paper	3.89 ± 0.26	4.74	0.85
Newspaper	3.14 ± 0.43	4.38	1.24

^1^ The paper acidity measured by the FL method are reported as the average of three determinations ± standard deviation.

## Data Availability

The data presented in this study are available on request from the corresponding author.

## Supplementary Materials

The following are available online at https://www.mdpi.com/article/10.3390/nano11071726/s1, Figure S1: The UV absorption spectra of the ethanol eluent of CdZnSeS-OA QDs after being treated by pATP, Figure S2: Normalized UV absorption spectra for CdZnSeS-OA QDs in n-Hexane, pATP and CdZnSeS-OA QDs after treatment by pATP ligand exchange in ethanol, Figure S3: The fluorescence spectra of HAc solution with different concentrations related to Figure 5c, Figure S4: (a) The fluorescence spectra of filter papers dried by extraction to variations of pH related to Figure 6c. (b) The fluorescence spectra of filter papers dried under CO_2_ to variations of pH related to Figure 6d, Figure S5: The variation of the fluorescence intensity at 537 nm with pH for CdZnSeS QDs on the paper, Figure S6: Normalized fluorescence lifetime decay curves for (a) CdZnSeS-OA QDs and CdZnSeS-pATP QDs and (b) CdZnSeS-pATP QDs under different pHs, Figure S7: (a) 1H NMR spectrum of QD-pATP supernate in D_2_O/DMSO-d6 mixture at a pH of 6. (b) 1H NMR spectrum of QD-pATP supernate in D_2_O/DMSO-d6 mixture at a pH of 1, Figure S8: (a) The fluorescence spectra of CdZnSeS-pATP QDs in the presence of NaCl, NaNO_3_ and Na_2_SO_4_ at different concentrations related to Figure 7a. (b) The fluorescence spectra of CdZnSeS-pATP QDs in the presence of gelatin-alum at different concentrations at a pH of 3 related to Figure 7b. (c) The fluorescence spectra of CdZnSeS-pATP QDs in the presence of lignin at different concentrations at a pH of 3 related to Figure 7b, Figure S9: The UV absorption spectra of lignin solutions at different concentrations at a pH of 3, Figure S10: (a) Normalized UV-vis absorption spectra of the paper samples. (b) The fluorescence spectra of the paper samples using CdZnSeS-pATP QDs as probes, Table S1: The acidity of HAc solutions detected by two methods, Table S2: The measured acidity of paper with different standardized pH values by different methods.
