# Supplementary material for: Quaternary Alloy Quantum Dots as Fluorescence Probes for Total Acidity Detection of Paper-Based Relics
Source: Nanomaterials (Basel). 2021 Jun 30;11(7):1726. doi: 10.3390/nano11071726 (PMC8308194; doi:10.3390/nano11071726)
Supplement: Supplementary file 1 [file nanomaterials-11-01726-s001.zip › nanomaterials-1274484-supplementary.pdf]

Quaternary Alloy Quantum Dots as Fluorescence Probes for Total Acidity Detection of Paper-Based Relics

Zhuorui Wang [1], Cong Cheng [1], Yongjuan Cheng [1], Lizhen Zheng [2,*], Daodao Hu [1,*]

[1] Engineering Research Center of Historical Cultural Heritage Conservation, Ministry of Education, School of Materials Science and Engineering, Shaanxi Normal University, Xi'an 710119, China
wangzhuorui@snnu.edu.cn (Z.W.); congcheng2017@snnu.edu.cn (C.C.); chengyongjuan@snnu.edu.cn (Y.C.); daodaohu@snnu.edu.cn (D.H.)

[2] School of Historical Culture and Tourism, Xi'an University, Xi'an 710065, China
lizhenzheng@snnu.edu.cn (L. Z.)

* Correspondence: lizhenzheng@snnu.edu.cn (L.Z.); daodaohu@snnu.edu.cn (D.H.)

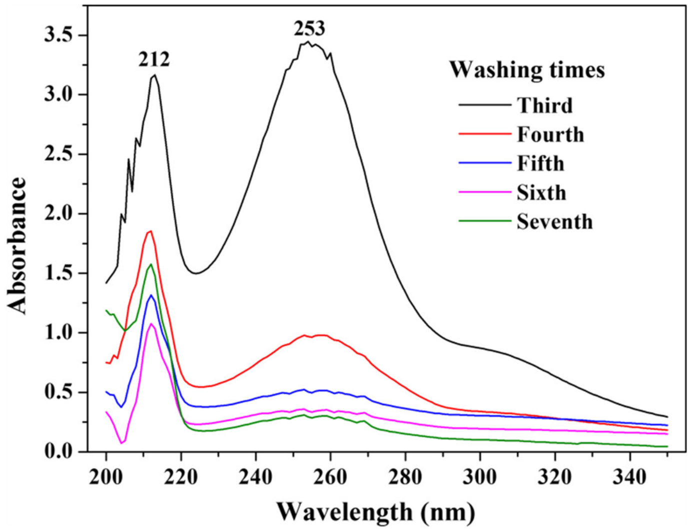

Figure S1. The UV absorption spectra of the ethanol eluent of CdZnSeS-OA QDs after treated by pATP.

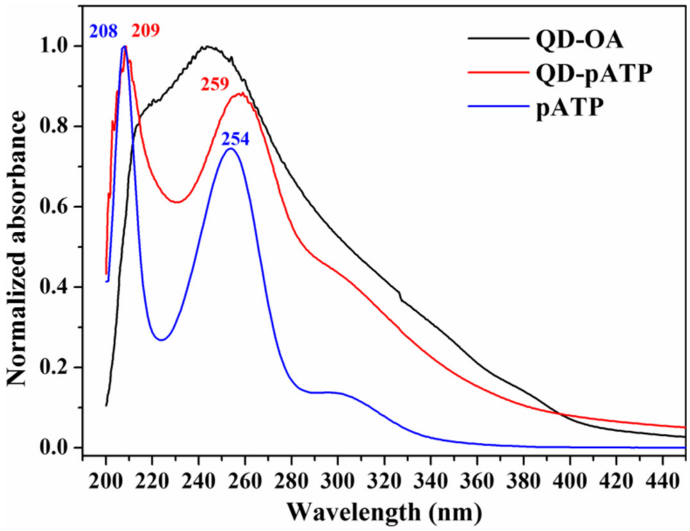

Figure S2. Normalized UV absorption spectra for CdZnSeS-OA QDs in n-Hexane, pATP and CdZnSeS-OA QDs after treatment of pATP ligand exchange in ethanol.

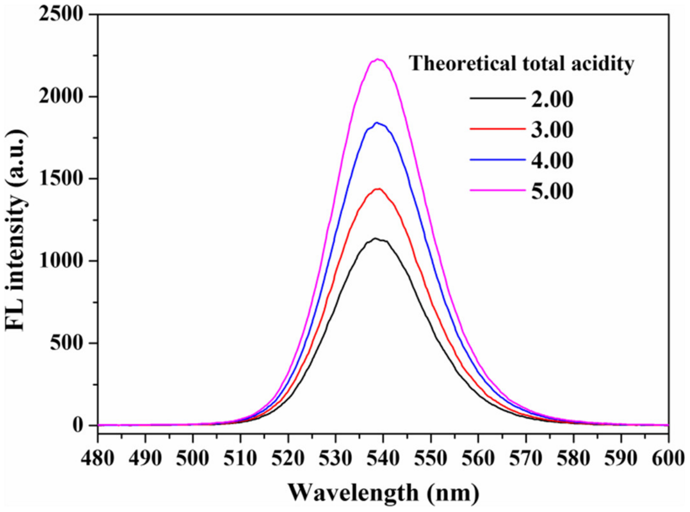

Figure S3. The fluorescence spectra of HAc solution with different concentrations related to Figure 5c. (C_{QDs}=8.28×10^{-5} mg/mL, Water:DMSO=150:1(V/V), λ_{ex}=365 nm).

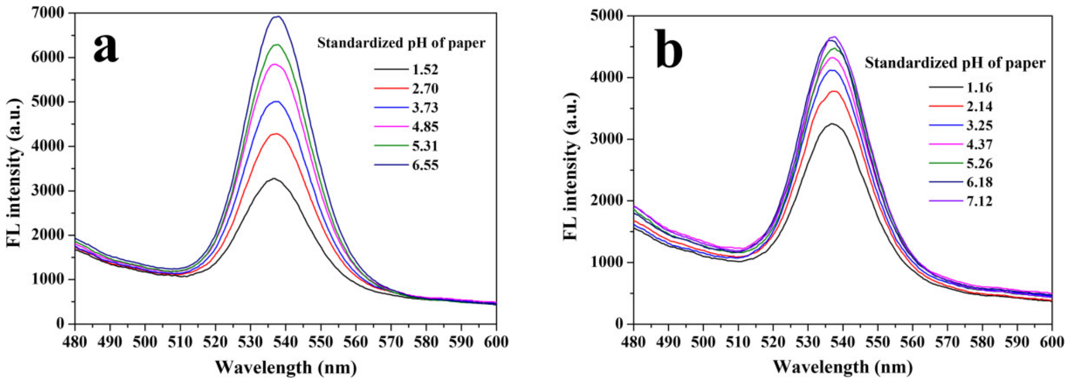

Figure S4. (**a**) The fluorescence spectra of filter paper dried by extraction to the variation of pH related to Figure 6c. (**b**) The fluorescence spectra of filter paper dried under CO_2 to the variation of pH related to Figure 6d. (λ_{ex}=365 nm).

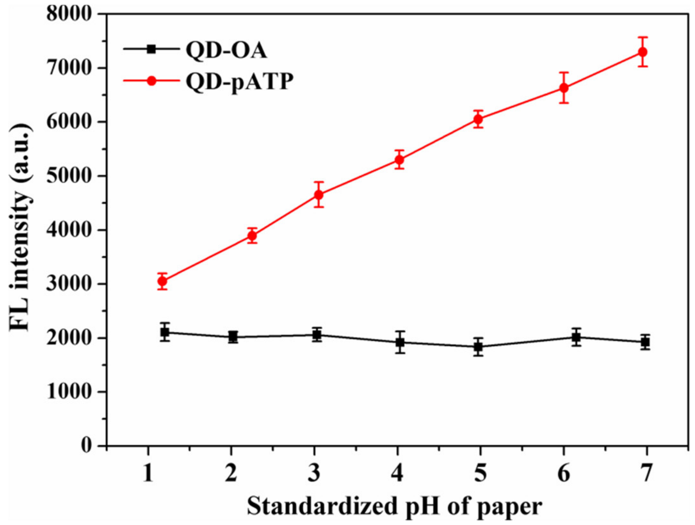

Figure S5. The variation of the fluorescence intensity at 537 nm with pH for CdZnSeS QDs on the paper. The error bars represent the standard deviations of four measurements. (λ_{ex}=365 nm).

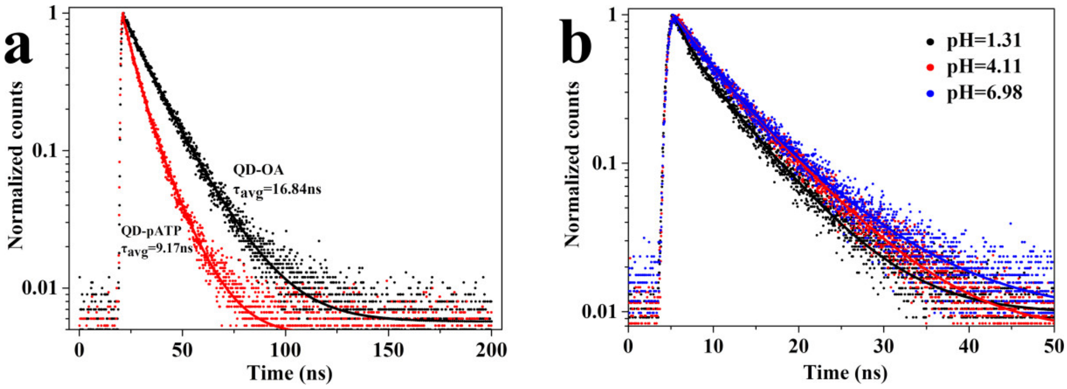

Figure S6. Normalized fluorescence lifetime decay curves for (**a**) CdZnSeS-OA QDs and CdZnSeS-pATP QDs and (**b**) CdZnSeS-pATP QDs under different pH. The lines represent the corresponding fitting curves. (λ_{ex}=340 nm, λem=537 nm).

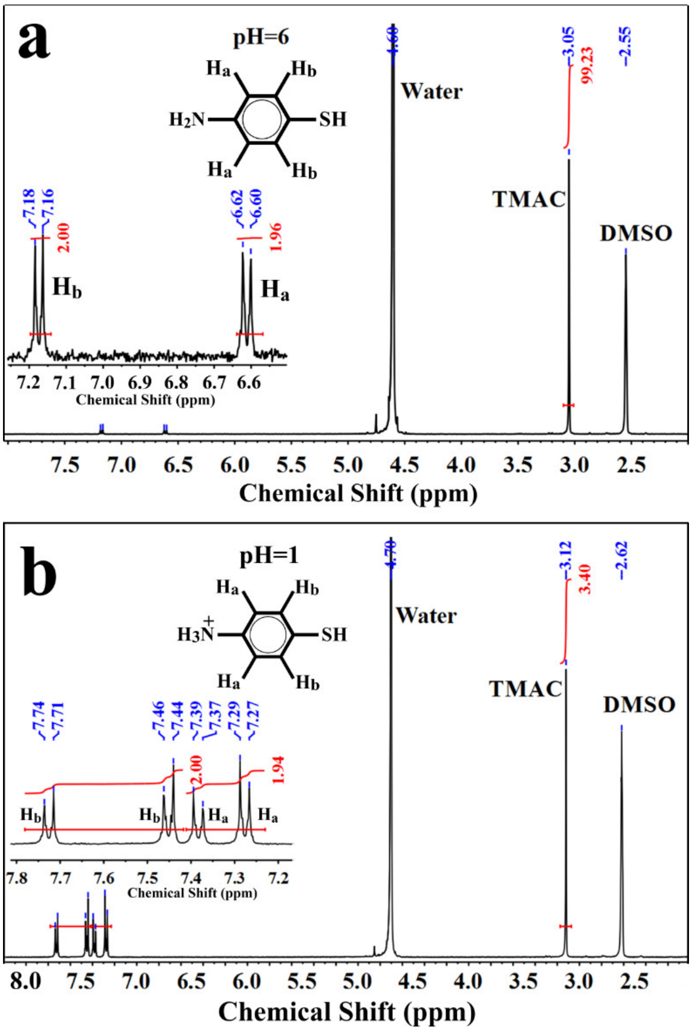

Figure S7. (**a**) ^{1}H NMR spectrum of QD-pATP supernate in D_2O/DMSO-d_6 mixture at pH of 6. (**b**) ^{1}H NMR spectrum of QD-pATP supernate in D_2O/DMSO-d_6 mixture at pH of 1. Insets show the enlarged spectra of protons in phenyl of pATP. Resonances are assigned in blue, and relative integrated peak areas are listed in red. The pH of suspension was adjusted with DCl. TMAC was used as internal standard substance with same concentration in both (**a**) and (**b**). (C_{QDs}=1.25 mg/mL, C_{TMAC}=1.02×10^{-5} M, D_2O:DMSO-d_6=1:1(V/V)).

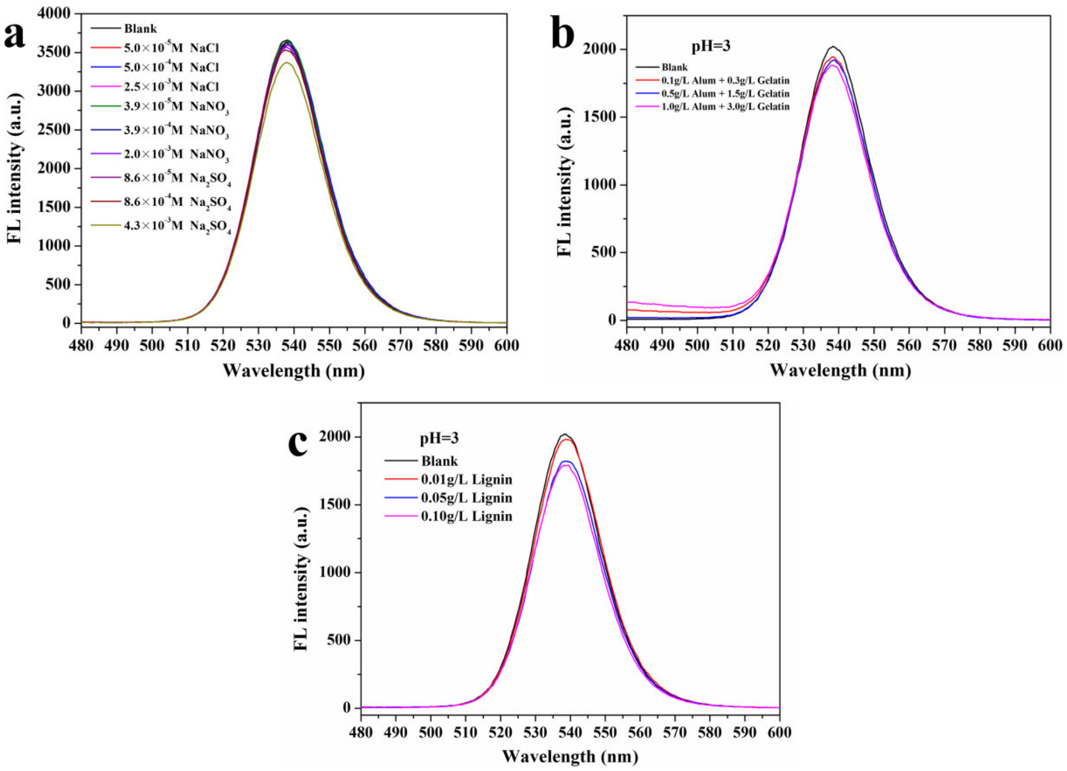

Figure S8. (**a**) The fluorescence spectra of CdZnSeS-pATP QDs in presence of NaCl, $NaNO_3$ and Na_2SO_4 at different concentrations related to Figure 7a. (**b**) The fluorescence spectra of CdZnSeS-pATP QDs in presence of gelatin-alum at different concentrations at pH of 3 related to Figure 7b. (**c**) The fluorescence spectra of CdZnSeS-pATP QDs in presence of lignin at different concentrations at pH of 3 related to Figure 7b. (C_{QDs}=1.15×10^{-4} mg/mL, Water:DMSO=150:1(V/V), λ_{ex}=365 nm).

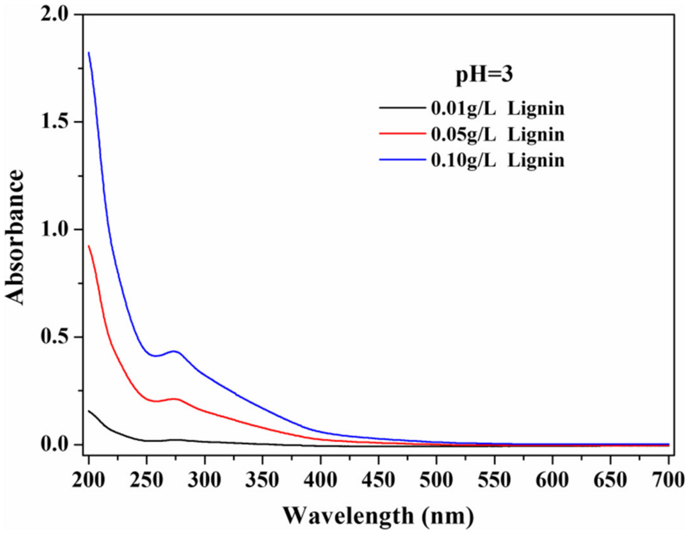

Figure S9. The UV absorption spectra of lignin solutions at different concentrations at pH of 3.

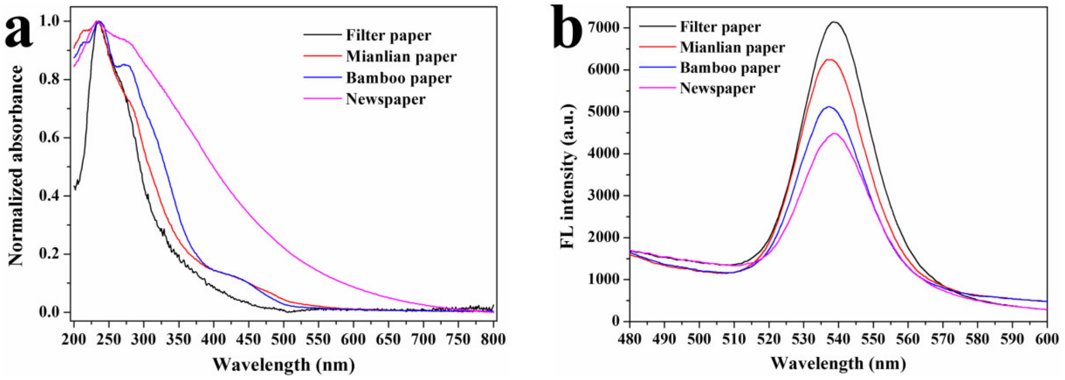

Figure S10. (**a**) Normalized UV-Vis absorption spectra of the paper samples. (**b**) The fluorescence spectra of the paper samples by using CdZnSeS-pATP QDs as probes. (λ_{ex}=365 nm).

Table S1. The acidity of HAc solution detected by two methods.

C_{HAc} (M)	Acidity Measured by pH meter	Acidity Measured by CdZnSeS-pATP QDs[1]	Theoretical Total Acidity
1.0×10^{-2}	3.33	2.45±0.10	2.00
1.0×10^{-3}	3.88	3.21±0.05	3.00
1.0×10^{-4}	4.48	4.20±0.11	4.00
1.0×10^{-5}	5.23	5.16±0.08	5.00

[1] The acidity of HAc solution measured by FL method are reported as average of four determination ± standard deviation.

Table S2. The measured acidity of paper with different standardized pH by different methods.

Standardized pH of paper	Acidity of Paper Dried by Extraction/Measured by FL Method [1]	Acidity of Paper Dried by Extraction/Measured by Extraction Method	Standardized pH of Paper	Acidity of Paper Dried under CO_2 /Measured by FL Method [2]	Acidity of Paper Dried under CO_2/Measured by Extraction Method
1.52	1.54±0.20	2.98	1.16	1.18±0.27	2.36
2.70	2.65±0.12	3.97	2.14	2.25±0.26	3.38
3.73	3.46±0.34	4.31	3.25	2.60±0.12	4.13
4.85	4.96±0.21	5.62	4.37	3.02±0.33	4.42
5.31	5.24±0.21	5.88	5.26	3.20±0.29	4.40
6.55	6.46±0.31	6.47	6.18	3.41±0.36	4.55
			7.12	3.37±0.19	4.77

[1,2] The paper acidity measured by FL method are reported as average of four determination ± standard deviation.
